# Supplementary material for: Kinome-wide Decoding of Network-Attacking Mutations Rewiring Cancer Signaling
Source: Cell. 2015 Sep 24;163(1):202–17. doi: 10.1016/j.cell.2015.08.056 (PMC4644236; doi:10.1016/j.cell.2015.08.056)
Supplement: Document S1. Supplemental Experimental Procedures [file mmc1.pdf]

Cell
Supplemental Information

Kinome-wide Decoding of Network-Attacking Mutations Rewiring Cancer Signaling

Pau Creixell, Erwin M. Schoof, Craig D. Simpson, James Longden, Chad J. Miller, Hua Jane Lou, Lara Perryman, Thomas R. Cox, Nevena Zivanovic, Antonio Palmeri, Agata Wesolowska-Andersen, Manuela Helmer-Citterich, Jesper Ferkinghoff-Borg, Hiroaki Itamochi, Bernd Bodenmiller, Janine T. Erler, Benjamin E. Turk, and Rune Linding

SUPPLEMENTAL EXPERIMENTAL PROCEDURES

Maintenance of Ovarian Cancer Cell Lines and Preparation of Sequencing Samples

ES2, OVAS, OVISE, TOV-21 and KOC-7C cells were grown in RPMI supplemented with 10% FBS and 1% PenStrep. The provenance of our cell lines was confirmed by STR analysis and is further detailed in the Figure S1. DNA was extracted from 2 x 10^6 cells at 80% confluency using a Qiagen QIAamp DNA Mini kit, according to the manufacturers instructions. The purified DNA was then sent to Roche Nimblegen for full exome (SeqCap EZ Human Exome Library v3.0 capture kit) sequencing. The sequencing results (FastQ files available at the Sequence Read Archive, SRA, under the study number PRJNA248664) were aligned using the Burrows–Wheeler Alignment Tool to the human reference genome (NCBI version GRCh37). After quality score recalibration and Indel realignment using the GATK package, SNP calling was performed with SAMtools. Next, we further filtered with VCFtools' default settings as well as a minimum coverage threshold of 10X in order to obtain high-quality variants. Finally, similarly as in the case of the global repository of somatic cancer mutations, the fasta files required by ReKINect containing both wild type and mutant protein sequences for all variants were generated using the Variant Effect Predictor (VEP) (Flicek et al., 2014) and purpose-made Python scripts.

Preparation of (Phospho-)Proteomics Samples

ES2, OVAS, OVISE, TOV-21 and KOC-7C cells were labeled with medium Stable Isotope Labeling by Amino acids in cell Culture (SILAC) and grown to ~80% confluency in sufficient 15cm dishes to provide 12mg of protein as starting material in triplicate. Each medium-labeled sample would be subsequently mixed 1:1 with a Spike-in SILAC (Geiger et al., 2011; Monetti et al., 2011) sample labeled with heavy SILAC containing a mix of peptides from the different ovarian cell lines, so that such internal standard would allow inter-sample comparisons. After cell cycle synchronization, by serum starvation for 24 hours, cells were lysed using ice-cold modified RIPA buffer supplemented with Roche complete protease inhibitor cocktail tablets and ß-glycerophosphate (5mM), NaF (5mM), Na-orthovanadate (1mM, activated). After cell lysis, samples were sonicated on ice, spun down (4,400g for 20mins at 4ºC) and proteins were precipitated over-night in ice cold acetone at -20ºC. Next, samples were dissolved in 6M Urea, 2M Thiourea, 10mM HEPES pH 8.0 and further reduced with 0.1mM DTT for one hour, alkylated with 5.5mM Chloroacetamide for another hour, after which they were pre-digested with Lysyl Endopeptidase at a 1:200 enzyme-to-protein ratio for four hours at room temperature. A dilution 1:4 with 50mM Ammonium Bicarbonate followed, after which trypsin was added at a 1:200 enzyme-to-protein ratio and left rotating over-night at room temperature. The following morning, the addition of TFA to a final concentration of 2% was used to suppress the enzymatic activity of trypsin, after which the samples were clarified and desalted using 360mg SepPak columns. Peptides were eluted twice with 2ml of 40% Acetonitrile, 0.1% TFA, and one final time with 2ml of 60% Acetonitrile, 0.1% TFA. For the phospho-peptide enrichment with Titanium Dioxide (TiO_2), the eluent was directly subjected to SCX fractionation and thus separated over a 30% SCX Buffer B (5mM potassium dihydrogen phosphate, 30% Acetonitrile, 350mM potassium chloride, pH2.7) gradient in 60 minutes at a 1ml/min flowrate. The resulting fractions were pooled according to their chromatographic properties and enriched for phosphorylated peptides, while specific aliquots were taken at this point for the global proteome analysis. The TiO_2 enrichment was conducted similarly to ref (Olsen et al., 2006), with several adjustments. For the TiO_2 loading solution, the TiO_2 beads were incubated in 0.02g/ml dihydrobenzoic acid dissolved in 30% Acetonitrile and 4% TFA for 15 minutes prior to peptide enrichment. 1.5mg of TiO_2 beads suspended in 6µl of TiO_2 loading solution was subsequently used to enrich each pooled SCX fraction and left to rotate end-over-end for 30 minutes at room temperature. The flow-through (i.e. early eluting fractions) was enriched three consecutive times. Samples were spun down at 2000g for 5 minutes and pelleted beads were washed with 100µl SCX Buffer B. After spinning the samples down again as before, beads were again pelleted and washed this time with 100µl 40% Acetonitrile, 0.25% acetic acid, 0.5% TFA. Finally, pelleted beads were re-suspended in 50µl 80% Acetonitrile, 0.5% acetic acid and transferred to separate C8 StageTips. Liquid was spun through at 2000g for 60s, after which the phosphorylated peptides were eluted with 1x 20µl 5% Ammonia and 1x 20µl 10% Ammonia, 25% Acetonitrile into a 96-well PCR plate, containing 20µl of 1% TFA, 5% Acetonitrile solution. Peptides were lyophilized to a total volume of 10µl, and acidified with 40µl of 1% TFA, 5% Acetonitrile, after which they were desalted on in-house packed C18 StageTips prior to LC-MS analysis.

For LC-MS analysis, peptides were eluted from the StageTip with 2x 20µl 80% Acetonitrile, 0.1% Formic acid, and lyophilized to 5µl final volume. The eluent was acidified with 1% TFA, 2% Acetonitrile and loaded onto a 50cm C18 EasySpray column, using the EasyLC 1000 UHPLC system (Thermo Fisher Scientific, USA). Peptides were eluted over a 250 minute gradient, ranging from 6-60% of 80% Acetonitrile, 0.1% Formic acid, and the Orbitrap Q Exactive (Thermo Fisher Scientific, USA) was run in a DD-MS2 top10 method. Full MS spectra were collected at a resolution of 70,000, with an AGC target of 3 x 10^6 or maximum injection time of 20ms and a scan range of 300-1750 m/z. The MS2 spectra were obtained at a resolution of 17,500, with an AGC target value of 1 x 10^6 or maximum injection time of 80ms. Dynamic exclusion was set to 20s, and ions with a charge state < 2 or unknown were excluded. For the proteome samples, the settings were the same, except for a gradient time of 230 mins, maximum MS2 injection time of 60ms and dynamic exclusion of 45s.

As shown in Figure S2, the Spike-In approach was done using a heavy-labelled SILAC reference sample coming from a proteomic mix of all the ovarian samples. All measurements where taken in biological triplicates and comparisons between same-sample and inter-sample confirmed the robustness of our method.

The samples were analyzed by liquid chromatography-mass spectrometry on an Orbitrap Q-Exactive (Thermo Fisher Scientific, USA). The mass spectrometry proteomics data have been deposited to the ProteomeXchange Consortium (http://www.proteomexchange.org) via the PRIDE partner repository (Vizcaíno et al., 2013) with the dataset identifier PXD000901.

Computational analysis of MS spectra

In order to investigate the effect of using mutant sample-specific fasta files as the MaxQuant search engine database, we performed the raw data searches using the wild-type ENSEMBL human FASTA (GRCh37/hg19) including all missense mutant proteins reported by sequencing. Further analysis and mapping of known phosphorylation sites to detect extinction of phosphorylation sites was done using in-house Python, R and MySQL scripts. MS search results were filtered on phosphorylation localization probability higher than 0.75 and MaxQuant peptide ID score higher than 50 in order to only use high confidence identifications.

Computing Minimum Distance to Substrate from PDB Files

As discussed in the main text, we included a measure of distance to the peptide substrate in our lists of mutations perturbing determinants of specificity. This distance was extracted by computing the distance between every residue in the kinase domain to every position in the peptide substrate for ten structures deposited in PDB, where kinase and substrate were co-crystallized (AKT2 [PDB ID: 1O6K]; (Yang et al., 2002), PIM1 [PDB ID: 2BZK]; (Debreczeni Bullock, A., Knapp, S., Von Delft, F., Sundstrom, M., Arrowsmith, C., Weigelt, J., and Edwards, A.), DYRK1A [PDB ID: 2WO6]; (Soundararajan et al., 2013), CDK2 [PDB ID: 2CCI]; (Cheng et al., 2006), PAK4 [PDB ID: 2Q0N]; (Filippakopoulos Eswaran, J., Turnbull, A., Papagrigoriou, E., Pike, A.W., Von Delft, F., Sundstrom, M., Edwards, A., Arrowsmith, C.H., Weigelt, J., and Knapp, S), EPHA3 [PDB ID: 3FXX]; (Davis et al., 2009), FES [PDB ID: 3CD3]; (Filippakopoulos et al., 2008), EGFR [PDB ID: 2GS6]; (Zhang et al., 2006), IGF1R [PDB ID: 1K3A]; (Favelyukis et al., 2001), INSR [PDB ID: 3BU3]; (Wu et al., 2008)). More specifically, with the use of in-house python scripts and a biopython package (Bio.PDB), we extracted all these distance features between all residues of these kinase-substrate pairs and collapsed the information about minimum distance observed with the kinase domain alignment. Any alignment position that could not be mapped to a structure was given the arbitrary maximum distance of 50Å.

Prioritization of Cancer Somatic Mutation Most Likely Causing Downstream Rewiring.

In order to prioritize the mutations identified by ReKINect as downstream rewiring, we integrated two extra sources of information. First, to prioritize based on the distance to the substrate peptide, we collected a comprehensive set of structures of protein kinases with a bound peptide substrate deposited in Protein Data Bank (PDB) (Berman et al., 2000) and computed the minimum distance between each alignment position and the peptide substrate (see Experimental Procedures). Additionally, using the dataset collected in our accompanying article (ref. Creixell et al. co-submitted article), we utilized information about whether previous methods had already identified the positions hit by these mutations as potential DoS, essentially providing independent validation of these results.

Table S1 provides the complete prioritized list of putative downstream rewiring NAMs, ordered by the likely contribution of the site to kinase specificity as measured by KINspect (specificity score, ref. Creixell et al. co-submitted article) and, where available, other sources in the literature as well as information about the average distance of residues in this kinase position to the substrate peptide (distance to substrate in Ångstrøms, Å).

In order to identify potential candidates for experimental validation, we prioritized our mutations on DoS based on their distance to substrate, their wild-type specificity having being previously experimentally determined and independent evidence of the determinants of specificity.

Protein Kinase Specificity Assays

Briefly, we used a 182-component peptide library with the general sequence Y-A-X-X-X-X-X-S/T-X-X-X-X-A-G-K-K(biotin), in which X postions were an equimolar mix of the 17 amino acid residues (excluding Cys, Ser and Thr), and S/T was an equal mix of Ser and Thr. Each component of the library had a single X position fixed as one of the 20 amino acids. Peptide mixtures (50 μM) were arrayed in 1536 well plates and incubated with kinase and ATP (50 μM including 0.03 μCi/μl γ−[^{33}P]ATP) for 2 hr at 30 ºC. Aliquots (200 nl) were transferred to a biotin capture membrane (Promega), which was washed, dried and exposed to a phospho screen as described (Mok et al., 2010). Spot intensities were quantified (QuantityOne software, BioRad) and normalized so that the average value within a position was assigned a value of 1. Data averaged from multiple runs were $\log_2$ transformed and used to generate heat maps. For PKCγ assays on individual peptide substrates, kinase (0.1 ug/ml) was incubated with 5 μM peptide in a buffer containing 50 mM Tris-HCl, pH 7.5, 10 mM $MgCl_2$, 1 mM DTT, 100 μM ATP with 0.3 μCi/μl γ−[^{33}P]ATP, and a 5-fold dilution of PKC lipid activator (provides diacylglycerol in phosphatidylserine vesicles and calcium, EMD-Millipore) for 15 min at 30°C. Aliquots were withdrawn at 5 min intervals and analyzed by P81 filter binding assay and scintillation counting to determine radiolabel incorporation (Hastie et al., 2006). Linear reaction rates were calculated from a standard curve generated from known quantities of radiolabel.

Phenotypic RNAi screening of Mutated Kinase and SH2 proteins

Cells were transfected with Silencer Select siRNAs (Life Technologies) using a 'one-step' method; siRNAs were diluted to 80nM in OptiMEM (Life Technologies) and mixed 1:1 with Lipofectamine RNAiMAX, also diluted in OptiMEM, such that each siRNA was mixed with 0.08μl of reagent. The siRNA/transfection reagent mix was then incubated at room temperature for 15 minutes prior to being dispensed into 384-well ViewPlates (PerkinElmer). Cell lines were plated directly into the siRNA containing wells at a density of 2000 (ES2), 3000 (KOC7C, TOV21 and OVISE) or 4000 (OVAS) cells per well. Cells were then incubated with the siRNAs for 72 hours at 37°C, 5% CO_2, 95% humidity before being fixed, stained and read on the Opera High Content Imaging reader (PerkinElmer). siRNAs were diluted 1 in 8 by the addition of cell culture medium giving a final, 'in-assay' concentration of 5nM. Cells were fixed by the addition of 4% paraformaldehyde (Sigma), incubated at room temperature for 15 minutes. Parafromaldehyde was then removed and cells were stained with Hoechst 33342 (Life Technologies) diluted to 2μg/ml in PBS. Cells were incubated for 1 hour, at room temperature, in the dark before being washed and imaged. Cells were imaged on the Opera using a x10 objective, 405nm laser excitation and 450/50 band pass emission filter. Nuclei were detected using the Acapella image analysis software (PerkinElmer).

Analysis of Phenotypic Changes Culminating from Genesis and Extinction of Phosphorylation Sites

Following perhaps the most parsimonious expectation, we hypothesized that the genesis of a phosphorylation site on TANC1 could lead to an observable phenotypic readout upon its knock-down and, on the contrary, the extinction of a phosphorylation site on RAB11FIP1 could decrease any pre-existing phenotypic impact upon knock-down. While knock-down effect could certainly be attributable to many other factors besides these specific mutations, surprisingly, we indeed observed a relatively small but robust ($P=1.7x10^{-4}$) loss of nuclear intensity upon knock-down of TANC1 only in the mutant cell line, OVAS (Figure S3E). Moreover, the decreased proliferation caused by the knock-down of RAB11FIP1 in cell lines wild-type for this gene is significantly ($P=3.1x10^{-3}$) reduced compared to the cell line harboring the mutant variant, KOC7C, most likely through a perturbation of cell cycle kinetics leading to an accumulation of cells in G1-phase (Figure S3D and F).

The statistical significance of changes in cell phenotypes caused by siRNA knockdown of ReKINect classified NAMs resulting in the generation or extinction of phosphorylation sites was evaluated using 1-way fixed effect ANOVA. The measured phenotypes were related to cell proliferation and included nuclei number and nuclei intensity (which changes throughout the cell cycle and during apoptosis). This analysis showed that the KOC7C cell line had a significantly different phenotype (in terms of nuclear proliferation) from those wild type for RAB11FIP1 (ES2, OVAS, OVISE and TOV21), $P=3.1x10^{-3}$. Conversely, the OVAS cell line had a significantly different phenotype (in terms of nuclear intensity) from those wild type for TANC1 (ES2, KOC7C, OVISE and TOV21), $P=1.7x10^{-4}$.

NAM based Regressor Models of Cell Proliferation

Changes in nuclei number were assayed, as described in Experimental Procedures, utilizing a library of 573 siRNAs (Ambion Silencer Select, Life Technologies) targeting kinase and SH2 domain containing genes.

Screens were performed in the ovarian cancer cell lines ES2, KOC7C, OVAS, OVISE and TOV21, and quantified data from triplicate repeats was normalized to negative i.e. non-targeting siRNA.

Of the 2865 tested conditions 314 caused a robust (greater than 2 standard deviations from the mean of the negative control) increase in nuclei number. A further 117 caused a robust decrease in nuclei number.

From these data we set out to predict changes in proliferation, Y, upon RNAi knockdown based on the number and types of NAMs in the neighborhood of target proteins as defined by the local protein-protein interaction (PPI) network, as detailed below.

The proliferation change for a knock-down target t and cell line c is denoted as Y_{tc}. The specific measurement uncertainty of Y pertaining to any knockdown and cell line was quantified using variation from the three biological repeats, denoted as σ_{tc}. In the following subsections we shall consider four different models, described in turn.

Baseline model

It was assumed that there was no correlation between proliferation and the PPI-neighborhood of the siRNA target. Consequently the global screen is simply regressed as:

$$Y_{tc}=k+e_{tc}, \quad e_{tc}\sim N(0; \sigma_{tc})$$

where k is a common constant for all knock-downs and cell lines, representing the baseline ($k\approx100$) and e_{tc} represents the uncertainty, which is assumed to be normally distributed with a standard deviation equal to the experimentally determined one, σ_{tc}.

Topology model

We also investigated whether the network topology in the vicinity of each siRNA target had an effect on proliferation. In this model the neighborhood of each target, t, and cell line, c, was characterized in terms of the number neighbors, $n^{(d)}_{tc}$ in the PPI-network at distance d=1 and d=2. The full model reads:

$$Y_{tc}=k+\Sigma_{d=1,2}\, a_N^{(d)}\cdot N^{(d)}_{tc}+e_{tc},$$

where $N^{(d)}_{tc}$ is a standardized version of $n^{(d)}_{tc}$, to ease the biological interpretation of the corresponding coefficients. Specifically, $N^{(d)}_{tc}=(n^{(d)}_{tc}-\mu_n^{(d)})/\sigma_n^{(d)}$, where $\mu_n^{(d)}$ and $\sigma_n^{(d)}$ are respectively the mean and standard deviation of the number of proteins at distance d from target, across all targets and cell lines. Consequently, a value of $a_N^{(d)}=1$ implies that if the number of proteins at distance d is one standard deviation larger than its mean, the proliferation is predicted to be one unit larger than baseline k, given that the other regressor is set to its mean. The model potentially comprises 3 independent regression coefficients (k, $a_N^{(1)}$, $a_N^{(2)}$).

Binary model

In this model, we examined whether the presence of a NAM in the vicinity of each siRNA target had an effect on proliferation. Specifically, we combined the topology model with the number of NAMs $m^{(d)}_{tc}$, in distance d=0, 1 and 2 away from the target, ignoring uninterpreted mutations. The full model reads:

$$Y_{tc}=k+\Sigma_{d=1,2}\, a_N^{(d)}\cdot N^{(d)}_{tc}+\Sigma_{d=0,1,2}\, a_M^{(d)}\cdot M^{(d)}_{tc} +e_{tc},$$

where $M^{(d)}_{tc}$ is a standardized version of $m^{(d)}_{tc}$, , c.f. the standardization procedure for $n^{(d)}_{tc}$. The model potentially comprises 6 independent regression coefficients (k, $a_M^{(0)}$, $a_M^{(1)}$, $a_M^{(2)}$, $a_N^{(1)}$, $a_N^{(2)}$).

Classified model

Finally, to assess whether the specific NAM classification provides further predictive power we derived an extension of the Binary model in which we distinguish between different uninterpreted and interpreted mutations (NAMs).

The targets addressed in the RNAi screen were classified using ReKINect alone without prior or additional knowledge and therefore entailed only four of the possible NAM classes (*downstream rewiring*, *extinction of phosphorylation site* , *node activation* and *node inactivation*) and an ‘uninterpreted’ class (i.e. where a mutation is not interpreted by ReKINect alone).
These classifications were, as to be expected, very unevenly distributed (downstream rewiring = 31, extinction of phosphorylation site = 377, node inactivation = 6, node activation=1, uninterpreted = 3856). Since some of the mutation counts were too low to be informative by themselves, we grouped mutations by categories (likely signaling activating or likely signaling inactivating) by combining node inactivating mutations with phosphorylation site extinctions, and similarly, combining node activations with downstream rewiring mutation. Thus, the full model reads:

$$Y_{tc}=k+\Sigma_{d=0-2}\, a_L^{(d)}\cdot L^{(d)}_{tc}+\Sigma_{d=0-2}\, a_R^{(d)}\cdot R^{(d)}_{tc}\ \Sigma_{d=0-2}\, a_U^{(d)}\cdot U^{(d)}_{tc}+\Sigma_{d=1,2}\, a_N^{(d)}\cdot N^{(d)}_{tc}+e_{tc},$$

where $L^{(d)}_{tc}$, $R^{(d)}_{tc}$ and $U^{(d)}_{tc}$ are the standardized (see above) number of mutations classified as either a loss of a phosphorylation site, downstream rewiring or uninterpreted, respectively, as function of the cell line c and distance d from the target t. Consequently, the model potentially comprises 12 independent regression coefficients (k, $a_L^{(0,1,2)}$, $a_R^{(0,1,2)}$, $a_U^{(0,1,2)}$, $a_N^{(1)}$, $a_N^{(2)}$). The conceptual idea of the classified (and NAM) model is illustrated in Figure S3A.

PPI-network
We based the network analysis on known physical PP-interactions as indexed by the combined score in the STRING resource (Szklarczyk et al., 2011). We pruned the network by removing all links with probabilities below a predefined cut-off value P_c, and assigning probabilities P=1 to the remaining links. The regression results listed below are for P_c=0.9, corresponding to what STRING terms high-confidence. The results, however, are insensitive to this choice, as verified by repeating the analysis with other cut-off values (0.5< P_c<0.9).

Variable and model selection
In order to avoid over-fitting we pruned away non-informative regressors in each of the considered models following a 2-step procedure. In the first step, a subset of regressors were selected using two different standard methods: AIC-optimization (Akaike, 1974) and shrinkage via the elastic-net (EN) regularization (Zou and Hastie, 2005). In the first approach, the subset of variables is found by minimizing the Akaike information criterion (AIC). Due to the low-dimensional nature of the parameter space, we have determined this set by an exhaustive search of all 2^D possibilities, where D is the number of coefficients, $a_X^{(d)}$ in the model. The second approach, EN-regularization, combines two traditional shrinkage techniques, LASSO- (Tibshirani, 1994) and Tikhonov-regularization (Tikhonov and Arsenin, 1978). The method is characterized by a single global parameter f ($0\le f\le 1$), which dictates the balance between the two types of regularization (f=0, Tikhonov and f=1, LASSO). For each value of f, we determine the overall regularization scale by minimizing the 10-fold cross-validation error using the efficient R source-package implementation “glmnet” (Friedman et al., 2010). As the cross-validation error turned out to be insensitive to the choice of f, a ‘mean’ EN-model was constructed by including only those regressors that appeared more than 50% of times in a scan of f from 0 to 1 in steps of 0.1.
Both the resulting AIC-model and the mean EN-model was subsequently pruned by keeping only those regressors in a standard linear regression that proved significantly different from zero (two-sided t-test, α=0.05).
The regression model with the best predictive power was then chosen as the final model.The regression results for these final versions of the NAM and classified models are given in Figure S3B, yielding a reduction to only 2 and 3 regressors, respectively, excluding the baseline.
The performance of the final classified model is illustrated in Figure S3C.

Model comparison
The four models were compared in terms of their respective AIC-score, root mean square residuals R, and P-values. AIC values for the baseline, topology model, binary model and classified model were 4606, 4587, 4554 and 4538 respectively. R values were 2.246, 2.238, 2.224 and 2.217 respectively. P-values were derived by ANOVA-model comparison.
Network topology (topology model), the presence of NAMs (binary model) and the classification of those NAMs (classified model) were all found to have significant predictive power compared to the baseline model with p-values of 4.8×10^{-6}, 7.1×10^{-13} and 6.3×10^{-16} respectively.

Cell-cycle Phase Analysis
Cell-cycle analysis by quantitation of DNA content was undertaken using flow cytometry. 72 hours after knockdown (as described in phenotypic RNAi screening of mutated Kinase and SH2 proteins, but scaled for 6-well plate format), cells were trypsinised and washed once in ice-cold phosphate-buffered saline (PBS) and counted. Cells were pelleted and then fixed by drop wise addition of ice-cold 70% ethanol while vortexing and incubated on ice for 30 minutes. Cells were then pelleted and washed twice with PBS before treatment with 50µl per 1×10^6 cells, of a 100µg/ml stock of DNase-free RNase for 15 minutes at 37’C. DNA was then stained by addition of 200µl per 1×10^6 cells, of 1µg/mL DAPI in PBS. Flow cytometry was carried out on an LSRII equipped with UV laser (BD Biosciences). Initial gating of cells was based on FSC-A and SSC-A. Doublets were excluded based on gating of FSC-A vs. SSC-W and DAPI-A vs. DAPI-W. Gated single cells were then plotted as a histogram using the DAPI-A parameter with standard gates defined for G1, S phase and G2M. Gates were defined on negative control populations and subsequently applied to corresponding knockdowns. Voltages were only adjusted between cell lines to facilitate placing of G1 populations on the same scale. All cell cycle analysis was carried out in biological triplicate for each of the cell lines and conditions.

Statistical significance of the shift in cell cycle kinetics and an accumulation of cells in G1 phase from G2/M and S phase was evaluated using a t-test. P-values for the wild type cell lines were $8.6x10^{-3}$, $1.3x10^{-2}$, $2.1x10^{-2}$ and $2.0x10^{-3}$ for ES2, OVAS, OVISE and TOV21 respectively. Conversely, the P-value for the mutant cell line, KOC7C, was $7.8x10^{-2}$ i.e. all wild type cell lines, and not the mutant cell line, showed a significant shift in cell cycle kinetics.

SUPPLEMENTAL REFERENCES

Akaike, H. (1974). A new look at the statistical model identification. IEEE Trans. Automat. Contr. *19*.

Berman, H.M., Westbrook, J., Feng, Z., Gilliland, G., Bhat, T.N., Weissig, H., Shindyalov, I.N., and Bourne, P.E. (2000). The Protein Data Bank. Nucleic Acids Res *28*, 235–242.

Cheng, K.Y., Noble, M.E., Skamnaki, V., Brown, N.R., Lowe, E.D., Kontogiannis, L., Shen, K., Cole, P.A., Siligardi, G., and Johnson, L.N. (2006). The role of the phospho-CDK2/cyclin A recruitment site in substrate recognition. J Biol Chem *281*, 23167–23179.

Davis, T.L., Walker, J.R., Allali-Hassani, A., Parker, S.A., Turk, B.E., and Dhe-Paganon, S. (2009). Structural recognition of an optimized substrate for the ephrin family of receptor tyrosine kinases. FEBS J *276*, 4395–4404.

Debreczeni Bullock, A., Knapp, S., Von Delft, F., Sundstrom, M., Arrowsmith, C., Weigelt, J., and Edwards, A., J.E. Crystal structure of the human Pim1 in complex with AMP-PNP and Pimtide.

Favelyukis, S., Till, J.H., Hubbard, S.R., and Miller, W.T. (2001). Structure and autoregulation of the insulin-like growth factor 1 receptor kinase. Nat Struct Biol *8*, 1058–1063.

Filippakopoulos, P., Kofler, M., Hantschel, O., Gish, G.D., Grebien, F., Salah, E., Neudecker, P., Kay, L.E., Turk, B.E., Superti-Furga, G., et al. (2008). Structural coupling of SH2-kinase domains links Fes and Abl substrate recognition and kinase activation. Cell *134*, 793–803.

Filippakopoulos Eswaran, J., Turnbull, A., Papagrigoriou, E., Pike, A.W., Von Delft, F., Sundstrom, M., Edwards, A., Arrowsmith, C.H., Weigelt, J., and Knapp, S, P. Structure of human p21 activating kinase 4 (PAK4) in complex with a consensus peptide.

Flicek, P., Amode, M.R., Barrell, D., Beal, K., Billis, K., Brent, S., Carvalho-Silva, D., Clapham, P., Coates, G., Fitzgerald, S., et al. (2014). Ensembl 2014. Nucleic Acids Res *42*, D749–D755.

Friedman, J., Hastie, T., and Tibshirani, R. (2010). Regularization Paths for Generalized Linear Models via Coordinate Descent. J. Stat. Softw. *33*, 1–22.

Geiger, T., Wisniewski, J.R., Cox, J., Zanivan, S., Kruger, M., Ishihama, Y., and Mann, M. (2011). Use of stable isotope labeling by amino acids in cell culture as a spike-in standard in quantitative proteomics. Nat Protoc *6*, 147–157.

Hastie, C.J., McLauchlan, H.J., and Cohen, P. (2006). Assay of protein kinases using radiolabeled ATP: a protocol. Nat. Protoc. *1*, 968–971.

Mok, J., Kim, P.M., Lam, H.Y.K., Piccirillo, S., Zhou, X., Jeschke, G.R., Sheridan, D.L., Parker, S.A., Desai, V., Jwa, M., et al. (2010). Deciphering protein kinase specificity through large-scale analysis of yeast phosphorylation site motifs. Sci Signal *3*, ra12.

Monetti, M., Nagaraj, N., Sharma, K., and Mann, M. (2011). Large-scale phosphosite quantification in tissues by a spike-in SILAC method. Nat Meth *8*, 655–658.

Olsen, J. V, Blagoev, B., Gnad, F., Macek, B., Kumar, C., Mortensen, P., and Mann, M. (2006). Global, in vivo, and site-specific phosphorylation dynamics in signaling networks. Cell *127*, 635–648.

Soundararajan, M., Roos, A.K., Savitsky, P., Filippakopoulos, P., Kettenbach, A.N., Olsen, J. V, Gerber, S.A., Eswaran, J., Knapp, S., and Elkins, J.M. (2013). Structures of Down syndrome kinases, DYRKs, reveal mechanisms of kinase activation and substrate recognition. Structure *21*, 986–996.

Szklarczyk, D., Franceschini, A., Kuhn, M., Simonovic, M., Roth, A., Minguez, P., Doerks, T., Stark, M., Muller, J., Bork, P., et al. (2011). The STRING database in 2011: Functional interaction networks of proteins, globally integrated and scored. Nucleic Acids Res. *39*.

Tibshirani, R. (1994). Regression Selection and Shrinkage via the Lasso. J. R. Stat. Soc. B *58*, 267–288.

Tikhonov, A.N., and Arsenin, V.Y. (1978). Solutions of Ill-Posed Problems. Math. Comput. *32*, 1320–1322.

Vizcaíno, J.A., Côté, R.G., Csordas, A., Dianes, J.A., Fabregat, A., Foster, J.M., Griss, J., Alpi, E., Birim, M., Contell, J., et al. (2013). The Proteomics Identifications (PRIDE) database and associated tools: status in 2013. Nucleic Acids Res. *41*, D1063–D1069.

Wu, J., Tseng, Y.D., Xu, C.F., Neubert, T.A., White, M.F., and Hubbard, S.R. (2008). Structural and biochemical characterization of the KRLB region in insulin receptor substrate-2. Nat Struct Mol Biol *15*, 251–258.

Yang, J., Cron, P., Good, V.M., Thompson, V., Hemmings, B.A., and Barford, D. (2002). Crystal structure of an activated Akt/protein kinase B ternary complex with GSK3-peptide and AMP-PNP. Nat Struct Biol *9*, 940–944.

Zhang, X., Gureasko, J., Shen, K., Cole, P.A., and Kuriyan, J. (2006). An allosteric mechanism for activation of the kinase domain of epidermal growth factor receptor. Cell *125*, 1137–1149.

Zou, H., and Hastie, T. (2005). Regularization and variable selection via the elastic net. J. Royal Stat. Soc. B *67*, 301–320.
