# Supplementary material for: Kinome-wide Decoding of Network-Attacking Mutations Rewiring Cancer Signaling
Source: Cell. 2015 Sep 24;163(1):202–17. doi: 10.1016/j.cell.2015.08.056 (PMC4644236; doi:10.1016/j.cell.2015.08.056)
Supplement: Document S2. Article plus Supplemental Information [file mmc11.pdf]

Cell

Resource

Kinome-wide Decoding of Network-Attacking Mutations Rewiring Cancer Signaling

Graphical Abstract

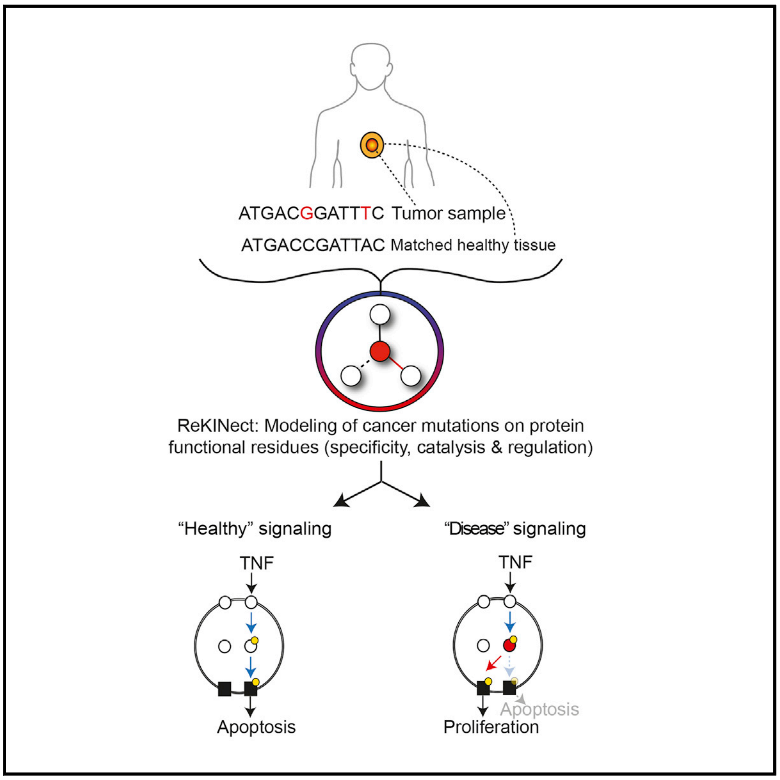

Authors

Pau Creixell, Erwin M. Schoof, Craig D. Simpson, ..., Janine T. Erler, Benjamin E. Turk, Rune Linding

Correspondence

linding@lindinglab.org

In Brief

A systematic classification of genomic variants in cancer reveals the many ways in which signaling networks can be perturbed, including rewiring and the creation or destruction of phosphorylation sites.

Highlights

- Mutations perturbing signaling networks are systematically classified and interpreted
- Several such functional mutations are identified in cancer and experimentally validated
- The results suggest that a single point mutant can have profound signaling effects
- Systematic interpretation of genomic data may assist future precision-medicine efforts

Creixell et al., 2015, Cell *163*, 202–217
September 24, 2015 ©2015 The Authors
http://dx.doi.org/10.1016/j.cell.2015.08.056

Resource

Kinome-wide Decoding of Network-Attacking Mutations Rewiring Cancer Signaling

Pau Creixell,[1,7] Erwin M. Schoof,[1] Craig D. Simpson,[2] James Longden,[2] Chad J. Miller,[3] Hua Jane Lou,[3] Lara Perryman,[2] Thomas R. Cox,[2] Nevena Zivanovic,[4] Antonio Palmeri,[5] Agata Wesolowska-Andersen,[1] Manuela Helmer-Citterich,[5] Jesper Ferkinghoff-Borg,[2] Hiroaki Itamochi,[6] Bernd Bodenmiller,[4] Janine T. Erler,[2] Benjamin E. Turk,[3] and Rune Linding[1,2,*]

[1]Department of Systems Biology, Technical University of Denmark, 2800 Lyngby, Denmark
[2]Biotech Research and Innovation Centre (BRIC), University of Copenhagen (UCPH), 2200 Copenhagen, Denmark
[3]Department of Pharmacology, Yale University School of Medicine, New Haven, CT 06520, USA
[4]Institute of Molecular Life Sciences, University of Zurich, 8057 Zurich, Switzerland
[5]Centre for Molecular Bioinformatics, University of Rome Tor Vergata, 00133 Rome, Italy
[6]Tottori University School of Medicine, Yonago 683-8504, Japan
[7]Present address: Koch Institute for Integrative Cancer Research, Massachusetts Institute of Technology (MIT), Cambridge, MA 02139, USA
*Correspondence: linding@lindinglab.org
http://dx.doi.org/10.1016/j.cell.2015.08.056
This is an open access article under the CC BY license (http://creativecommons.org/licenses/by/4.0/).

SUMMARY

Cancer cells acquire pathological phenotypes through accumulation of mutations that perturb signaling networks. However, global analysis of these events is currently limited. Here, we identify six types of network-attacking mutations (NAMs), including changes in kinase and SH2 modulation, network rewiring, and the genesis and extinction of phosphorylation sites. We developed a computational platform (ReKINect) to identify NAMs and systematically interpreted the exomes and quantitative (phospho-)proteomes of five ovarian cancer cell lines and the global cancer genome repository. We identified and experimentally validated several NAMs, including PKCγ M501I and PKD1 D665N, which encode specificity switches analogous to the appearance of kinases de novo within the kinome. We discover mutant molecular logic gates, a drift toward phospho-threonine signaling, weakening of phosphorylation motifs, and kinase-inactivating hotspots in cancer. Our method pinpoints functional NAMs, scales with the complexity of cancer genomes and cell signaling, and may enhance our capability to therapeutically target tumor-specific networks.

INTRODUCTION

Since the discovery of the first oncogene, Src (Stehelin et al., 1976), and tumor suppressor, Rb (Friend et al., 1986), more than three decades ago, our understanding of some of the specific genetic aberrations supporting cancer progression has steadily risen. Recent advances in next-generation sequencing technologies have led to the identification of large numbers of somatic cancer mutations through whole genome and exome sequencing of tumors. Given how complex it is to assess the relevance of this enormous repertoire of reported somatic cancer mutations (currently running in excess of 1 million variants) (Forbes et al., 2011), the discovery of new somatic mutations has vastly outpaced our ability to unravel their functional roles (Figure S1A).

Despite the fact that alterations to the physiological cellular responses to environmental cues are fundamental hallmarks of cancer cells (Hanahan and Weinberg, 2000) and that cellular responses to input cues are driven by signaling networks, a comprehensive understanding of how mutations perturb these networks is still missing. In fact, new conceptual paradigms and computational strategies allowing better assessment of the intrinsic complexities of cancer cells, such as the integration of cancer genomic and proteomic data, have been recently pinpointed as key requirements in the field of cancer research (Weinberg, 2014; Yaffe, 2013). Specifically, new approaches for decoding mutations that perturb signaling networks (or as we term them, ''network-attacking'' mutations [NAMs]) (Creixell et al., 2012a) and the mechanisms by which they may statically or dynamically alter these networks will be fundamental in closing this gap (Figure S1B) (Yaffe, 2013). Here, we describe and validate such a conceptual and computational framework capable of identifying, classifying and unraveling the impact of numerous predicted NAMs.

RESULTS

Classifying Mutations Affecting Signaling Networks

In order to evaluate whether cancer mutations perturb signaling networks, we initially developed a classification system with concrete types of NAMs. We divide NAMs into three fundamental classes.

The first and relatively well-described type of NAM is one that disrupts signaling network dynamics by constitutively activating or inactivating a protein kinase, thereby maintaining the

information flow either “on” or “off” uninterrupted over time. Examples of such “on” mutations are those substitutions that mimic activation loop phosphorylations, whereas examples of “off” mutations include those that alter catalytically essential residues of kinases, or residues in SH2 domains that are critical for phospho-tyrosine binding. Since the timely activation and termination of signals is critical for the proper cellular homeostasis as well as phenotypic responses to environmental stimuli, such mutations lead to aberrant information processing (Figure 1A).

A second, largely undescribed type of NAMs are those mutations that shift the signaling network structure by “rewiring” upstream or downstream interactions (of the mutated protein or node). Upstream rewiring can be caused by mutations in a kinase substrate that disrupt the linear motif around a phosphorylation site, thus causing a new upstream kinase to phosphorylate the mutant substrate. Downstream rewiring, in contrast, can be caused by drifts in the peptide specificity upon mutation of the determinants of specificity (DoS) in kinase (or SH2) domains (Creixell et al., 2015 [this issue of *Cell*]) (Figure 1A).

Finally, we hypothesized that a third type of NAMs could exist where mutations would generate or destroy phosphorylation sites, effectively generating new molecular logic gates in cancer cells (Figure 1A).

Node inactivation and node activation would fall within the categories of what is traditionally referred to as loss-of-function and gain-of-function hypermorphic mutations, while the other mutations would fit best within a gain-of-function neomorphic classification.

The ReKINect Methodology

With the aim of systematically identifying NAMs in phosphorylation-based signaling networks, we developed a computational approach, ReKINect, capable of predicting these defined functional mutations (Figures 1A and 1B; http://ReKINect.science). We began by assembling comprehensive sequence and positional information covering all known 538 kinase domains, 111 SH2 domains, and 149,838 phosphorylation sites in the human proteome (refer to the Experimental Procedures for further information). This information facilitated the mapping of NAMs onto these domains and the modeling of the likely functional effect of mutations (Figure 1B). Mutations in established or predicted functional residues (essential residues on the different domains, determinants of specificity identified in our accompanying paper [Creixell et al., 2015] as well as phosphorylation sites) would then be predicted to lead to the dysregulation of network dynamics, network rewiring, and gain or loss of phosphorylation sites (Figure 1B).

Below, we provide an overview, further details, and experimental evidence using a wide range of techniques (including genome-specific global phospho-proteomics, peptide specificity, or phenotypic data) for the different predictions generated by the ReKINect algorithm and explore the impact on signaling networks of the NAMs we identify.

Quantifying NAMs in Cancer Repositories and Cell Lines

Having defined the different NAMs, we next intended to assess their existence and abundance in cancer. We thus collected a set of 678,050 unique missense somatic cancer variants from COSMIC (version 67) (Forbes et al., 2011) and deployed ReKINect on this set to predict a large number of instances across the NAM classes (Figure 2).

In order to experimentally investigate NAMs, we performed a global integrative analysis by combining exome next-generation sequencing (NGS) and quantitative mass spectrometry (MS)-based (phospho-)proteomics on a set of five ovarian cancer cell lines (ES2, OVAS, OVISE, TOV-21, and KOC-7C; Figures S1 and S2) and conducted genome-specific proteomics analyses (Experimental Procedures). By following a Spike-in SILAC-based labeling strategy (Geiger et al., 2011) (Figures S1 and S2; Experimental Procedures), we could identify and accurately quantify on average more than 6,000 unique phosphorylation sites across over 2,000 proteins in each of the five cell lines. Furthermore, NGS identified close to 9,000 unique missense variants per cell line (including SNPs and germline mutations as well as somatic mutations) that were subsequently interpreted by ReKINect (Figure 2).

As shown in Figure 2 (and Data S1–S6) ReKINect could identify functional mutations covering each class of NAM included in our model as well as enrichments in these functional mutations (Figure S1). In addition, we computed the frequency at which different protein domains are affected by cancer mutations in the global repository of somatic cancer mutations as a means to provide general estimations of the likelihood of finding perturbations in different modular protein domains in cancer (Figure S1).

Given our currently limited knowledge about the different processes that can lead to the different NAMs (e.g., phosphomimicking mutations are the only case currently covered by ReKINect that result in kinase activation) the number of functional mutations presented in Figure 2 is most likely a significant underestimation. Nevertheless, in the following sections we provide further details and evidence supporting the existence of these predicted NAMs in cancer signaling networks.

Genesis and Extinction of Phosphorylation Sites and Circuitry

Having collected both exome sequencing and proteomic data on the same set of cancer cell lines, we were able to address the question of whether mutations could create new phosphorylation sites or destroy existing ones, thereby generating new cancer-associated molecular logic gates within a cancer cell signaling circuitry. To identify such events, we specifically inquired the global sequencing data for the appearance of phosphorylatable residues resulting from mutations, some of which could be experimentally verified to be bona fide sites by mass spectrometry. Strikingly, this approach uncovered several examples of mutations that lead to the genesis of new phosphorylatable sites, which become recognized and phosphorylated by kinases (Figures 3A and S2). Among the proteins harboring these neomorphic phosphorylation sites were TANC1 and HSF1 (Figure 3A). While little is known about TANC1, HSF1 is a heat-shock protein previously reported to be associated with carcinogenesis and poor prognosis, as well as supporting malignancy in a variety of cancers (Dai et al., 2007). Thus, further investigations of this new phosphorylation site on HSF1 and its

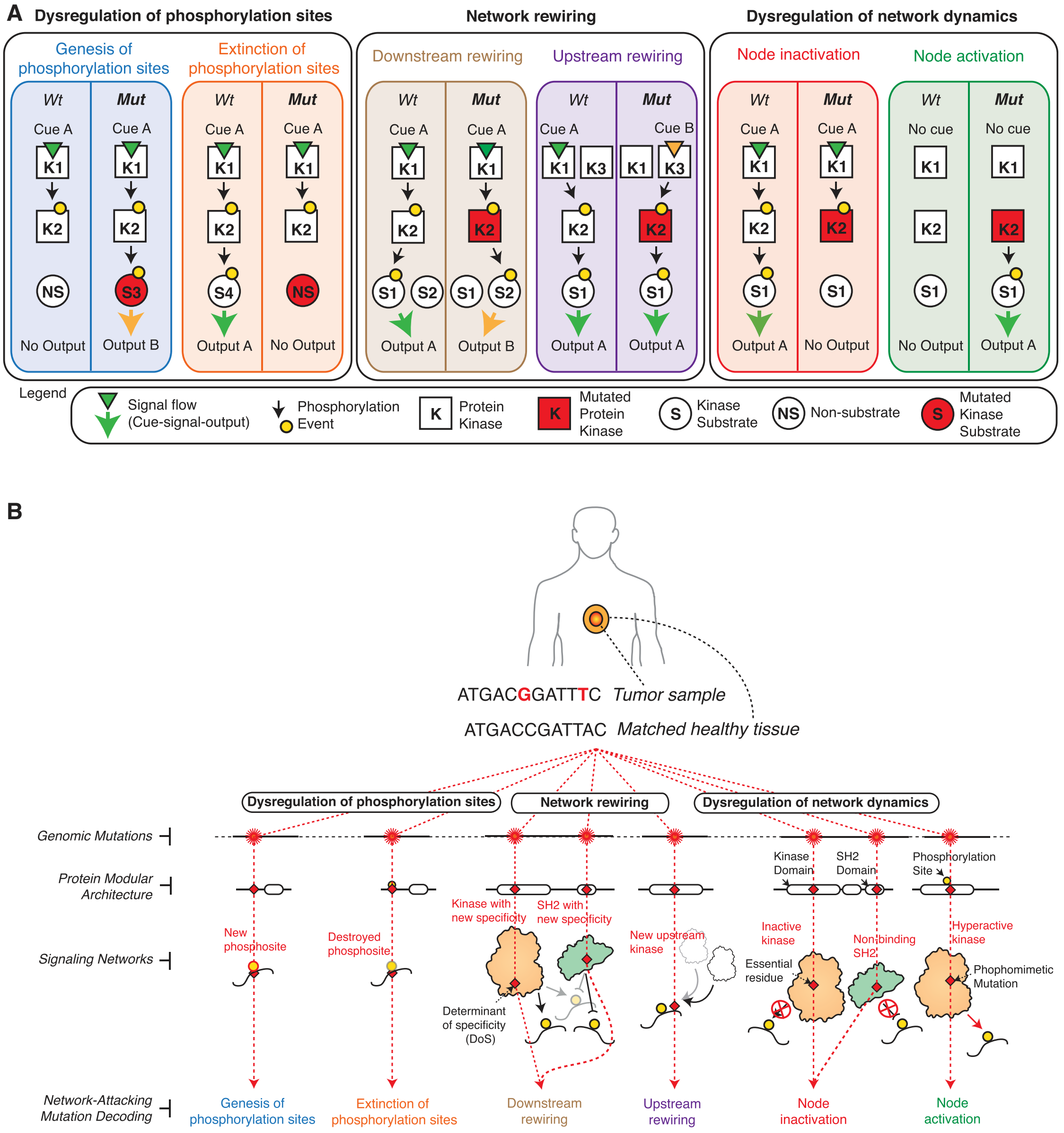

Figure 1. Network-Attacking Mutations

(A) Six distinct types of network-attacking mutations (NAMs) can be defined based on perturbations of signaling network dynamics, network structure, and dysregulation of phosphorylation sites. Cancer mutations could generate or destroy molecular logic gates, for example by creating new, or by removing existing, phosphorylation sites. Alternatively, mutant proteins could become activated by new upstream proteins (incoming edges) or start perturbing new downstream substrates (outgoing edges). Finally mutations could turn signaling proteins (e.g., protein kinases) constitutively "on" or "off." The effect of these NAMs on the cue-signal-output flow of information is illustrated for each comparing the wild-type (WT) and mutant (Mut) cases.

(B) After mapping mutations at the genomic and proteomic level, every NAM class defined in (A) is modeled on the different protein domains and motifs currently included in ReKINect following a distinct procedure: mutations on the essential residues of the kinase and SH2 domains are classified as node inactivating. Acidic mutations mimicking the phosphorylated/active state of kinases are classified as node activating. Mutations perturbing phosphorylation motifs and causing changes in the upstream kinase phosphorylating the target protein are classified as upstream rewiring. On the other hand, mutations in residues that determine specificity of the kinase or SH2 domains (Creixell et al., 2015) perturb domain specificity and are classified as downstream rewiring. Finally, our genome-specific MS experiments enable the identification of mutations generating phosphorylatable residues or the extinction of phosphorylation sites by mutating away from phosphorylatable residues.

See also Supplemental Experimental Procedures.

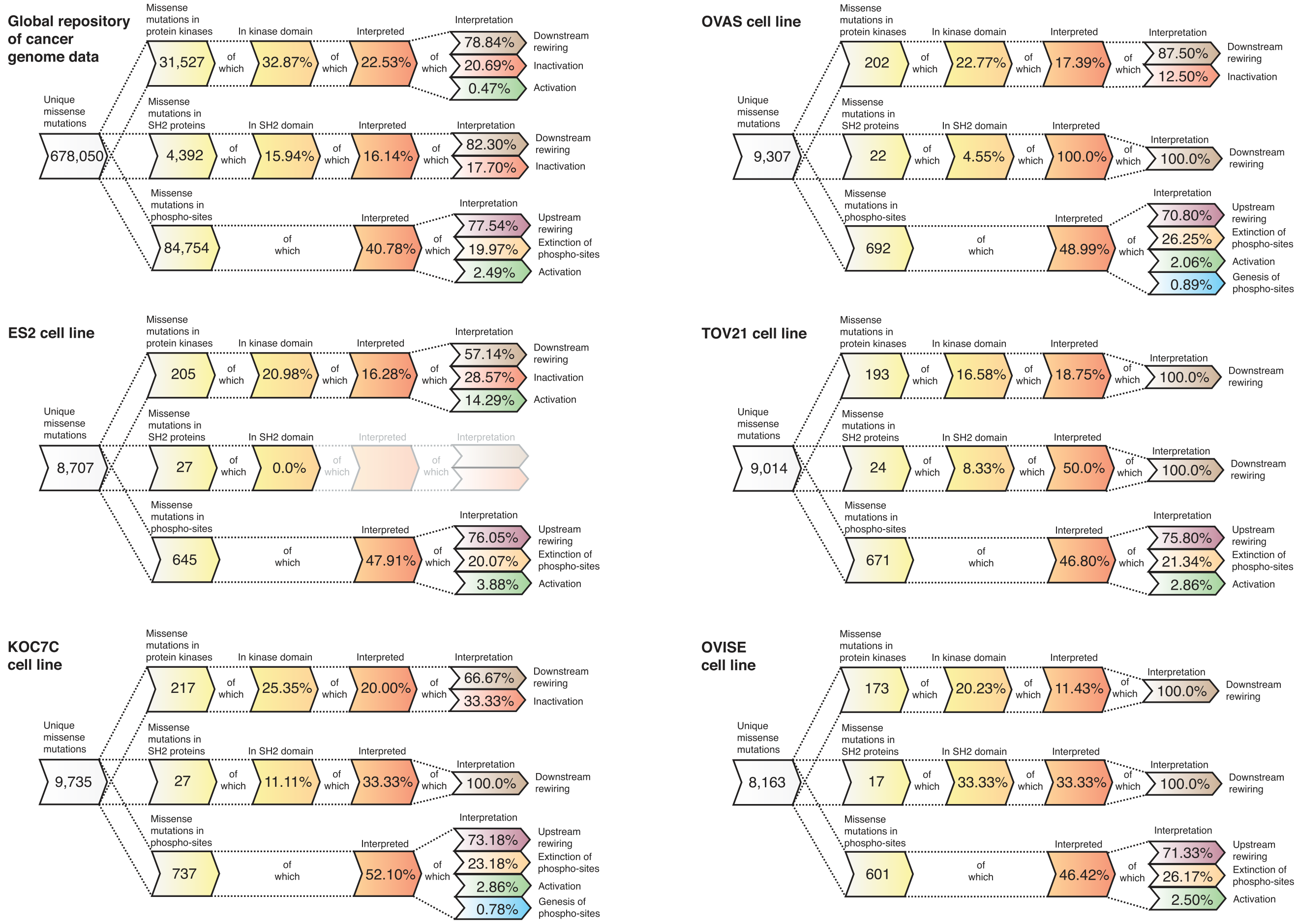

Figure 2. Overview of NAMs in Cancer Cell Lines and in the Global Repository of Cancer Somatic Mutations as Predicted by ReKINect

For each cell line and for the global repository of cancer somatic mutations we show the number of unique missense variants and how many of these variants fall within kinase proteins, SH2 proteins or phosphorylation sites (using a five-residue flanking region window surrounding the phosphorylation site). From these we then illustrate the fraction of variants falling within the respective domains and the fraction that can be interpreted by ReKINect. In the case of ES2, all of the 27 variants hitting an SH2 protein, hit outside SH2 domains, thus ReKINect could not make any predictions as to their effect (ghosted). It should be noted that the genesis of phosphorylation sites cannot be predicted from in silico analysis alone but require genome-specific-MS experiments. See also Figure S1.

predicted cell-cycle-dependent upstream kinase, CDK2, may lead to new insights on the role of this heat-shock protein in cancer (Figure 3A).

In order to discover NAMs destroying phosphorylation sites, we combined our exome sequencing data with those from the quantitative mass-spectrometry analysis of the phospho-proteomes of the five ovarian cancer cell lines. This enabled us to perform genome-specific searches of the mass-spectra, in order to identify direct proteomic evidence of the destruction of phosphorylation sites (Figure 3B) by identifying the mutated but unmodifiable peptides. This approach enabled us to identify 380 variants in our five cell lines and 6902 in the global repository of cancer mutations destroying phosphorylation sites (Experimental Procedures and Figure 2).

Two such events from the cell lines illustrated in Figure 3B, are RAB11FIP1 (T281M) and TNKS1BP1 (S1533G). Whereas the role of RAB11FIP1 in cancer is not as clear, Tankyrase-1-binding protein (TNKS1BP1) binds Tankyrase, which in turn, associates with TRF1 protein at the telomeres. This complex is not only tightly regulated during cell-cycle progression but critically it regulates telomere length by binding on the double-stranded TTAGGG repeat of telomeres. This, together with the fact that Aurora Kinase B (AurKB), a key cell-cycle mitotic kinase (Alexander et al., 2011), is predicted by NetworKIN (Linding et al., 2007) to phosphorylate the wild-type form of TNKS1BP1, suggests a potential role in cell-cycle and telomere length dysregulation for this mutant variant.

In order to provide further characterization and assess the phenotypic impact of mutations resulting in genesis and destruction of phosphorylation respectively, we performed siRNA-based knockdown experiments of both TANC1 and RAB11FIP1 across the five cell lines. While knockdown effect could certainly be attributable to many other factors besides these specific mutations, surprisingly, as shown in Figure S3 and detailed in the Supplemental Experimental Procedures, we indeed observed phenotypic effects supporting

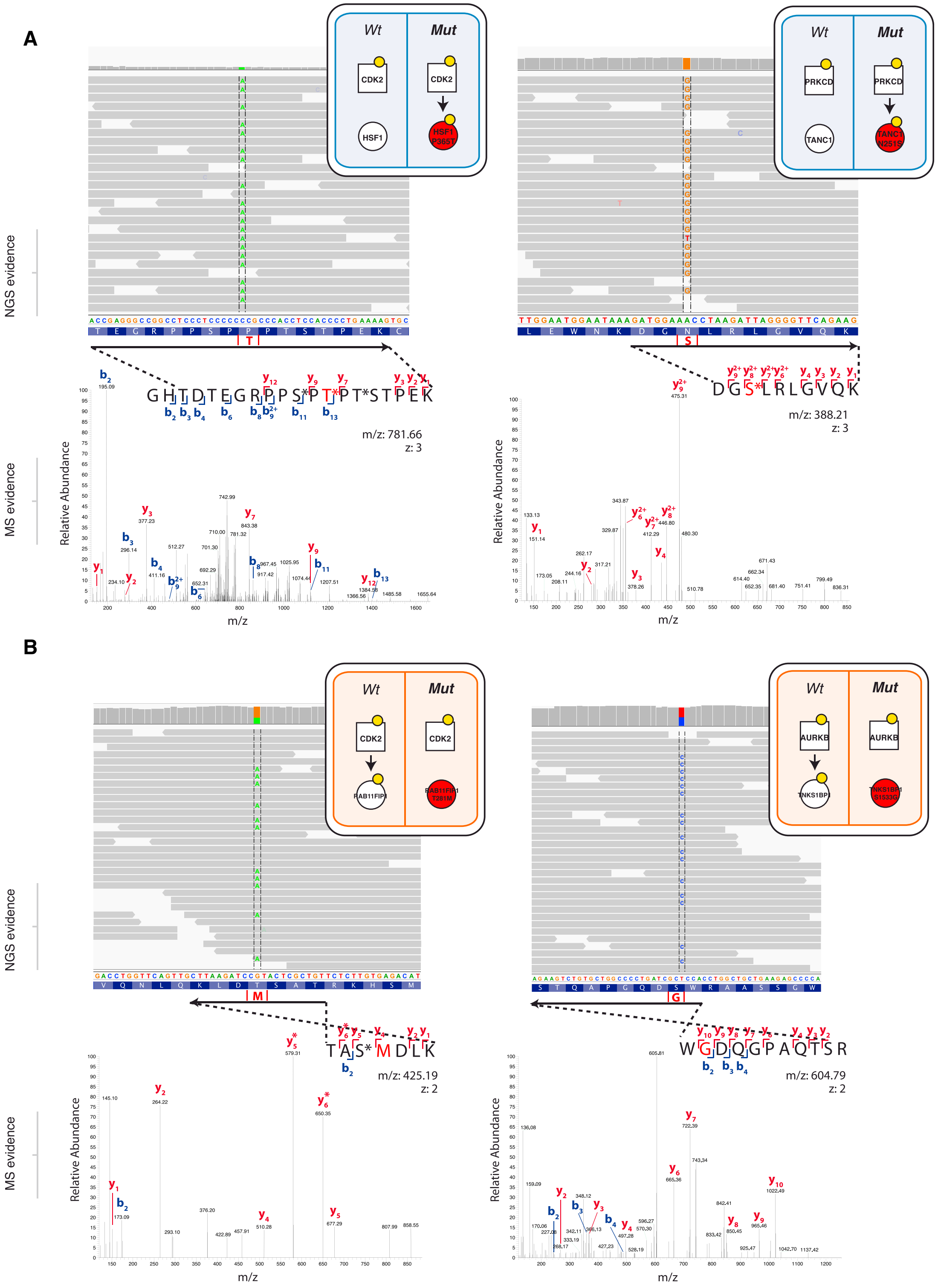

Figure 3. NAMs Leading to Genesis and Extinction of Phosphorylation Sites

(A) Two examples of network-attacking mutations generating new phosphorylation sites on HSF1 and TANC1, as evidenced by exome sequencing data and MS spectra matching the phosphorylated mutation.

(B) Two examples of network-attacking mutations causing the extinction of known phosphorylation sites on RAB11FIP1 and TNKS1BP1, supported by exome-sequencing data and MS spectra matching the unphosphorylatable mutated residue.

See also Figures S2 and S3.

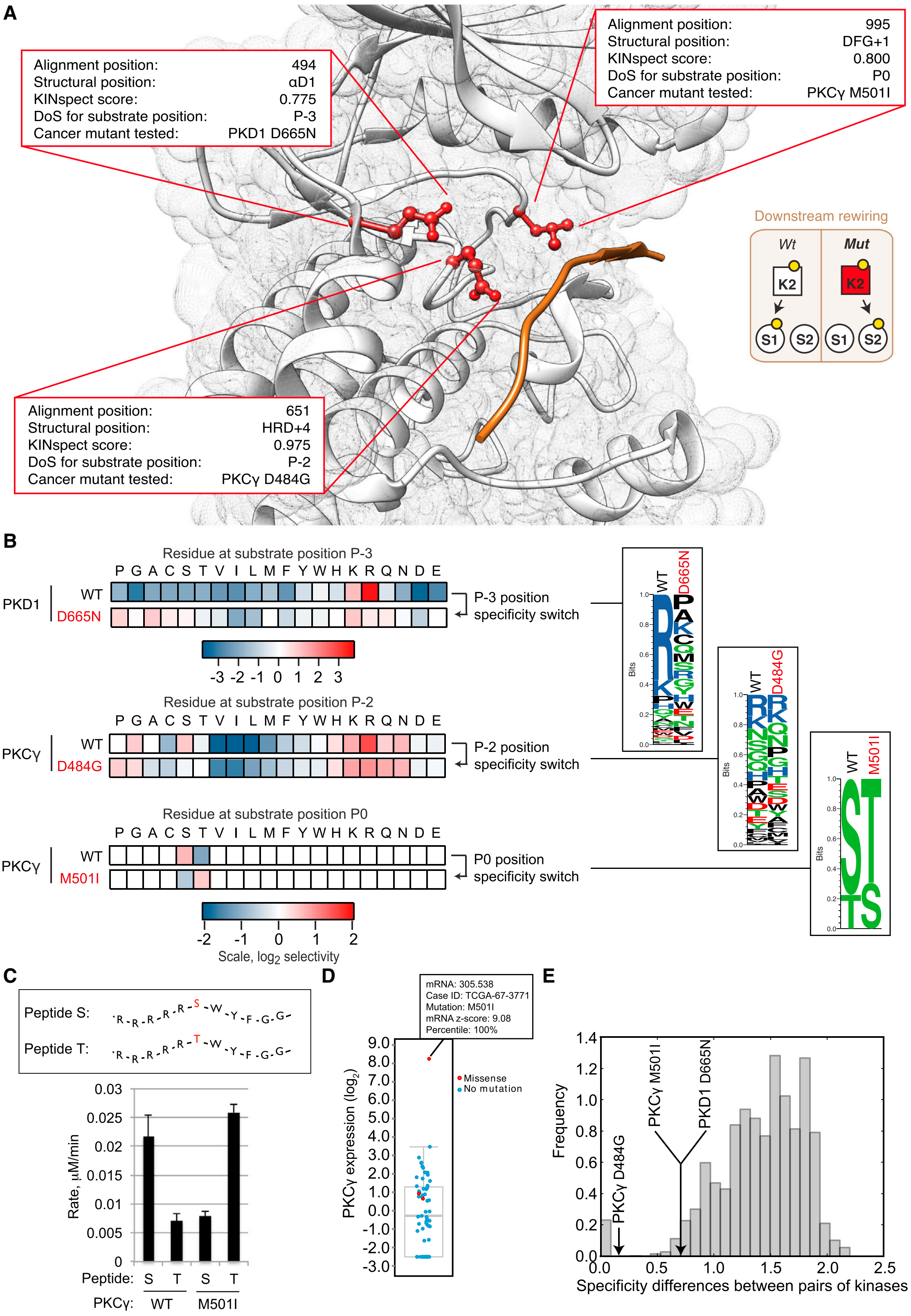

(legend on next page)

the most parsimonious expectations arising from ReKINect's predictions.

Above, we aimed to provide the most accurate and evident instances of NAMs that generate and destroy phosphorylation sites and achieved this through integration of exome-sequencing and MS experimental data following stringent selection criteria (Experimental Procedures). Thus, we speculate that many more NAMs leading to the genesis and extinction of molecular logic gates will undoubtedly exists.

Kinase Downstream Rewiring

Next, in order to explore if cancer mutations can hit residues that determine kinase specificity (determinants of specificity [DoS]) and thereby impose downstream rewiring, we included the results from the KINspect algorithm, described in the accompanying article (Creixell et al., 2015), in the ReKINect platform. Sourcing from the global repository of cancer-associated somatic mutations we could predict a large set of putative NAMs leading to downstream rewiring (Experimental Procedures; Table S1).

Following a prioritization procedure described in the Supplemental Experimental Procedures, we compiled a ranked list of cancer somatic mutations with the highest potential to cause downstream rewiring (Table S1). The list includes 1,871 unique missense mutations predicted to alter determinants of specificity by hitting the kinase domain residues most likely to play significant roles in specificity (specificity score higher than 0.9). Even with maximum stringency filters and focusing on the single kinase position most likely to drive specificity (highest specificity score of 1.0, previously reported by the literature as a determinant of specificity [Brinkworth et al., 2003] and in direct physical contact with the substrate with a distance of <3 Å), we identified 42 unique missense mutations on this specific position covering all branches of the human kinome tree (Table S1).

As detailed in the Supplemental Experimental Procedures, identifying the cases more suitable to experimental validation narrowed our candidates down to mutations on three positions in direct contact with the substrate and high KINspect score (Creixell et al., 2015) leading to the cloning, expression, and purification of these mutant kinases as well as their wild-type variants (Figures 4A–4E). First, we purified the two PKCγ mutants, D484G and M501I, predicted to perturb the determinants of specificity in alignment positions 651 and 995, respectively (Figures 4A and 4B). Since the determinant of specificity perturbed by the mutant variant D484G was located four residues downstream of the conserved HRD motif on the kinase domain, we named this determinant as HRD+4 (Figure 4A). Given this spatial location and proximity to the P−2 position of the substrate peptide, we predicted this first mutant would affect P−2 specificity. In contrast, the mutant variant M501I was found immediately downstream of the conserved DFG motif within the kinase activation loop (DFG+1), a residue for which there is recent evidence for its role driving serine-threonine specificity at the phosphorylation site (P0 i.e., central S/T(/Y) residue) position (Chen et al., 2014). As shown in Figures 4B and S4, experimental determination of the peptide specificity of both variants by positional scanning peptide library (PSPL) (Hutti et al., 2004) corroborated the specificity drift of both these mutants. In the case of the variant PKCγ D484G, our results uncovered a loss of Arg preference in position P−2 of the substrate peptide for the mutant variant (Figures 4B and S4). As predicted in the case of the variant M501I, PSPL results demonstrated a change in phosphoacceptor residue preference from Ser to Thr (Figure 5B). This specificity "switch" was further confirmed by performing phosphorylation assays on both the wild-type and mutant variants using a pair of matched peptide substrates of identical sequence save for having Ser or Thr in the P0 position (Figure 4C). As seen with PSPL analysis, WT PKCγ preferred Ser over Thr, while the M501I mutant by contrast phosphorylated the Thr peptide most efficiently. Given that PKCγ is a critical regulator of migration in development (Kramer et al., 2002), that it has been linked to metastasis (Yang et al., 2014), and that its overexpression in epithelial cells triggers a malignant phenotype and tumorigenic behavior in vivo (Mazzoni et al., 2003), we speculate that these specificity drifts ReKINect has predicted could provide tumorigenic, invasive, and metastatic capabilities to cancer cells. While these PKCγ mutants were identified in lung cancer samples (Kan et al., 2010) wild-type PKCγ is typically expressed only in the brain (Sundram et al., 2011). Interestingly, PKCγ was overexpressed in the tumor bearing the M501I mutation (Figure 4D) to levels substantially higher than in tumors where this genomic region had been amplified (as reported by cBioPortal [Gao et al., 2013]). A recent report highlighted loss-of-function mutations on PKC kinases

Figure 4. NAMs Causing Downstream Rewiring

(A) Three positions in direct contact with the substrate peptide, named αD1, HRD+4, and DFG+1, and likely involved in determining specificity for substrate positions P−3, P−2, and P0 (i.e., the phospho-acceptor site), respectively, harbor several cancer somatic mutations, three of which were selected for experimental validation.

(B) Experimental validation by position scanning peptide library (PSPL) array of the specificity drift caused by downstream rewiring NAMs. Heat maps show normalized, averaged data from two independent experiments illustrating the specificity drift for the cancer variants PKD1 D665N and PKCγ D484G and M501I in substrate positions P−3, P−2, and P0, respectively. The results are also shown in logo form plotting the normalized information content in the wild-type and mutant specificity switch position (logos generated using Seq2Logo [Thomsen and Nielsen, 2012]).

(C) The P0 specificity switch of the PKCγ variant M501I was subsequently confirmed by quantifying the phosphorylation rate of identical peptide substrates containing either Ser or Thr at the phosphorylation site position (RRRRRSWYFGG and RRRRRTWYFGG) by mutant and wild-type kinase variants. The graph shows the mean ± SD (n = 4).

(D) PKCγ expression levels are markedly increased in the tumor sample harboring the PKCγ M501I downstream rewiring mutation.

(E) Comparison of the differences in substrate specificity typically observed between wild-type human kinases (gray histogram) and those mutant kinases reported here (black arrows). As evident from the plot, in two out of the three cases, the magnitude of the specificity drift caused by the cancer mutations is comparable to the specificity difference existing between different wild-type kinases.

See also Figure S4.

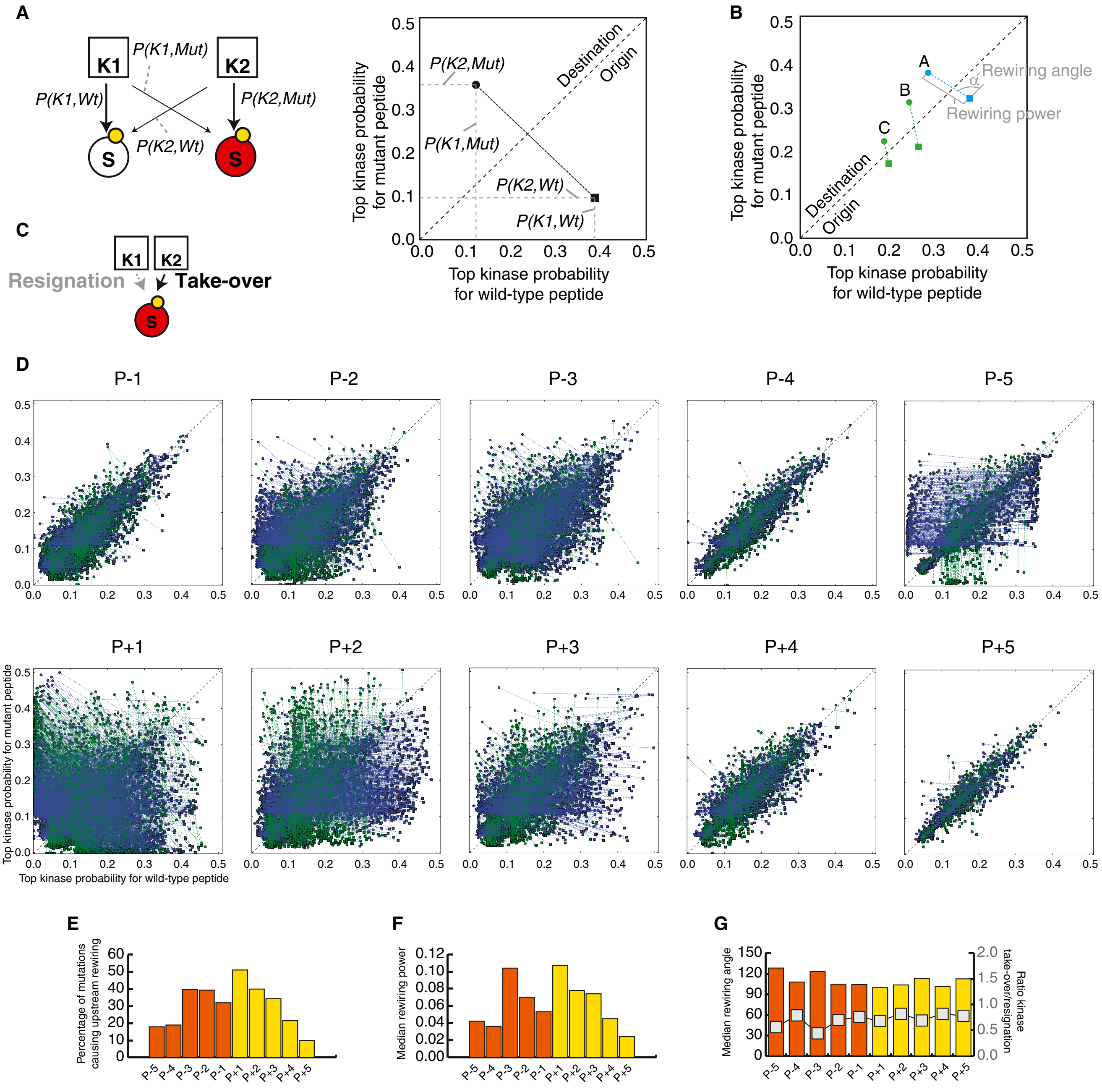

Figure 5. NAMs Causing Upstream Rewiring

(A) Upstream rewiring mutations will cause a new kinase (from K1 to K2) to phosphorylate the mutant protein (S, red). By plotting the probability of both kinases to phosphorylate the wild-type and mutant variants of the protein, we can visualize, quantify, and compare different upstream rewiring mutations.

(B) The rewiring power and the rewiring angle can be computed by considering the necessary trajectory that the mutation causes (from the "origin" right-bottom triangle to the "destination" left-top triangle). The rewiring power is equivalent to the magnitude of the vector and measures the rewiring capacity of the mutation. The rewiring angle is the angle of the vector from the diagonal and distinguishes whether the rewiring effect is mainly driven by kinase resignation (i.e., a loss of phosphorylating ability of the wild-type kinase, angle >45°), depicted in blue, or by kinase take-over (i.e., an increase of phosphorylation ability of a new kinase, angle <45°), depicted in green. The three examples illustrate how three different mutations (A–C) can lead to different outcomes, such as the same rewiring power but different main driving force (A and B) or the same driving force but different magnitude (B and C).

(C) Illustration of the two main driving processes that cause upstream rewiring, namely the reduced ability of the original kinase to phosphorylate the new mutant substrate variant (resignation) and the increased ability of a second kinase to phosphorylate the mutant substrate protein (take-over).

(D) Representation of all the upstream rewiring mutations identified in the global repository of somatic mutations at different distances relative to the phosphorylation site (from five residues before a phosphorylation site, P−5, to five residues after a phosphorylation site, P+5). Rewiring events mainly driven by resignation are shown in blue and those mainly driven by take-over are shown in green.

(legend continued on next page)

(Antal et al., 2015), including PKCγ. By having altered substrate specificity, the two PKCγ mutants characterized here are likely to both lose the ability to phosphorylate some endogenous substrates while gaining the capacity to phosphorylate new de novo substrates.

Next, we purified the mutant variant of PKD1 predicted to perturb the determinant of specificity in alignment position 494 (Figures 4A and 4B) that we named αD1, given its location on the first residue of α helix D of the kinase domain. As with the PKCγ mutants, the PSPL experiments validated the specificity drift in PKD1 D665N (Figures 4B and S4). Specifically, this mutation causes loss of an essential feature of the WT kinase phosphorylation signature, namely selectivity for Arg at the P−3 position. An Arg residue is found at the P−3 position in critical targets of PKD1, including CREB, SSH1L, HDACs 5 and 7, HPK1, MARK2, and HSP27. This variant is therefore expected to perturb signaling downstream of PKD1, a kinase with roles in the development and metastatic progression of several cancers including prostate, breast, gastrointestinal, pancreatic, and skin cancers (Sundram et al., 2011). Having made this prediction for a mutation originating from a prostate cancer sample (Lindberg et al., 2013), potential deregulation between PKD1 and its substrate HSP27 is particularly notable, as its phosphorylation is closely related to androgen receptor function in prostate cancer (Hassan et al., 2009; Sundram et al., 2011). In addition to breaking these interactions in the signaling network, because the D665N mutation renders PKD1 a less specific kinase, we anticipate that the mutation will generate many new connections through phosphorylation of non-native substrates, some of which may contribute to the malignant phenotype.

We next assessed the magnitude of the specificity switches caused by these cancer mutations, by comparing the wild-type to mutant drift in specificity to the specificity differences observed between wild-type human kinases across the kinome. As shown in Figures 4E and S4, two out of the three downstream rewiring mutations cause a specificity drift of a magnitude comparable to the specificity difference that exists between different wild-type human kinases. Effectively, this implies that a single cancer mutation can lead to a specificity switch that is analogous to a new kinase appearing in the genome.

With these validated examples at hand, we set out to further investigate whether other cancer mutations could cause similar dramatic specificity drifts and switches in other human kinases. By analyzing predictions from ReKINect based on cancer mutations identified to hit validated DoS residues (Table S1), in many cases with amino acid substitutions analogous to the ones we experimentally tested above, we could indeed identify additional cancer mutations that with high likelihood cause downstream rewiring. In the case of the HRD+4 site, 41 additional cancer mutations were identified substituting this site to multiple other residues (Table S1).

Moreover, in addition to the PKCγ M501I mutant, we could identify 29 other cancer mutations hitting the DFG+1 site, eight of which with analogous substitutions of large hydrophobic residues with β-branched aliphatic residues (Haspin L669I, DDR1 M793I, ITK M503V, TRKA M671T, IRAK3 M314I/M341V/M341T, and BRAF L597V), the type of substitution that most likely leads to a specificity switch from a preference for phosphorylating Ser to Thr (Figure S4; Table S1). In contrast, no mutant was found that would perturb specificity in the opposite direction (from Thr to Ser phosphorylation preference; Figure S4). Thus, it appears there is a general trend toward increased phosphoThr-driven signaling in cancer.

Of these 29 mutants, the identification of a likely mechanism of action for BRAF L597V is of critical relevance as it is not only a germline mutation in Noonan syndrome and cardio-facio-cutaneous syndrome, but also plays a significant role in the development of cancer when acting in epistatic synergy with Ras G12V (Andreadi et al., 2012; Davies et al., 2002). While the molecular mechanisms of this epistatic interaction could potentially be linked to changes in BRAF dimerization, our results suggest that Ras G12V could ensure the hyperactivity of this signaling network, whereas BRAF L597V rewires it by a drift in BRAF's kinase specificity. Such a scenario is reminiscent of previous interactions between different mutations promoting cancer development in a synergistic manner (Creixell et al., 2012a; Wu et al., 2010). Finally, we could identify 40 cancer mutations in addition to the PKD1 D665N mutation perturbing the αD1 site, eight of which containing the same amino acid substitution D to N (PKCb D427N, TSSK1 D97N, TTBK1 D116N, CDK11b D507N, CDK8 D103N, PFTAIRE1 D198N, PDGFRa D681N, and STYK1 D201N) and thereby constituting high-confidence downstream rewiring mutants (Figure S4; Table S1).

Altogether, these results represent the discovery of three new downstream rewiring mutations on three distinct determinants of specificity (HRD+4, DFG+1, and αD1) and show that single-point NAMs can drive downstream rewiring of a magnitude that is analogous to a new kinase suddenly appearing within the human kinome. They also suggest that the prioritized collection of mutations we provide is likely to contain even more cancer mutations causing rewiring (16 of which being clear high-confidence candidates, Figure S4).

Upstream Kinase Rewiring

Complementary to the downstream rewiring NAMs, we next investigated whether mutations could also cause upstream rewiring (i.e., when a substrate is, due to the impact of a mutation, being phosphorylated by different upstream kinases) by perturbing phosphorylation motifs on the substrate (Figure 1). By analyzing mutations that fall within 5 flanking residues of known phosphorylation sites (see Experimental Procedures) with the NetPhorest (Miller et al., 2008) and NetworKIN (Linding et al., 2007) algorithms on the wild-type and mutant variants of the

(E) Quantification of the percentage of mutations leading to upstream rewiring depending on their position relative to the phosphorylation site.
(F) Assessment of the median magnitude of rewiring for mutations based on their position relative to the phosphorylation site.
(G) The median rewiring angle (orange and yellow bars) and the ratio of take-over over resignation rewiring mutations (gray line) conditioned on the position of the mutation relative to the phosphorylation site.
See also Figure S5.

same protein, we could predict the likely upstream rewiring effects of mutations on substrates. As detailed in Figure 5 (see also Figure S5 and Tables S2 and S3), for a given predicted rewiring event (i.e., where the upstream kinase predicted for the wild-type and mutant variants of the substrate is non-identical) we defined two variables termed ‘‘rewiring power’’ and ‘‘rewiring angle’’ based on the predicted probability for the most likely upstream kinase in the wild-type and mutant substrate variants (Figures 5A and 5B).

The rewiring power measures the magnitude of the rewiring event, by accounting for the loss of phosphorylation potential of the old upstream kinase as well as the gain in phosphorylation potential of the new upstream kinase. The number of rewiring events and their rewiring power showed a non-uniform distribution where, generally, mutations closer to a phosphorylation site have a higher chance of causing upstream rewiring and the rewiring event itself will be of higher magnitude (rewiring power) (Figures 5D–5F). This global trend is observed for all positions except for the position just before the phosphorylation site, P−1, where mutations are less likely to lead to rewiring events and will most often be of lower magnitude. In fact, such distribution with the singularity of P−1 resembles the positional distribution of information content of kinase substrate specificity (Figure S5), underlining a fundamental link between the criticality of a given position for substrate recognition by upstream kinases and the disruptive potential of cancer somatic mutations hitting those positions. In other words, positions critical to and in direct close contact with the phosphorylating kinase (e.g., P+1, P+2, P−2, or P−3, as opposed to P−1 that makes very few contacts with the kinase) (Brinkworth et al., 2003) are far more likely to harbor strongly rewiring mutations.

The repertoire of potential upstream rewiring events allowed us to address the central question of whether rewiring is most often driven by an increased phosphorylation propensity for the mutant substrate variant by a new kinase (which we term ‘‘kinase take-over’’) or, inversely, if caused by a reduced propensity for the original kinase upon mutation (which we term ‘‘kinase resignation’’) (Figure 5C). The rewiring angle does, in effect, measure which of the two forces is stronger, with rewiring events mainly driven by kinase take-over leading to a rewiring angle <45° from the diagonal in Figure 5B, while rewiring events mainly driven by kinase resignation would be associated with rewiring angles >45°. As shown in Figure 5G, our results based on the median rewiring angle as well as the ratio of take-over/resignation events measured at different positions relative to the phosphorylation site show that, regardless of the position, upstream rewiring events are predominantly driven by kinase resignation forces. Illustrative examples of this can be found in Tables S2 and S3, where many of the most strongly rewiring events are caused by cancer mutations disrupting, for instance, proline residues in P+1 positions of CDK substrates. For example, a mutation juxtaposed to a phosphorylation site on position 721 of damage-specific DNA binding protein 1 (DDB1 P721Q) is predicted to cause an upstream shift from CDK1 to ATM and a similar mutation on CCP110 (CCP110 P171L) leads to a predicted upstream rewiring from CDK1 to PLK1 (Table S3). Finally, mutations on ORC1 (P312S), CDC23 (P583T), and NUMA1 (P113H) illustrate how disruption of pleiotropic recognition motifs, such as the one for CDK1 kinase, can lead to upstream rewiring events.

Overall, these results suggest that cancer mutations may rewire upstream signaling typically by worsening an optimal substrate site for a given upstream kinase and not by generating a more optimal substrate better matching another upstream kinase. Considering the fact that it has taken millions of years to evolve exquisitely fine-tuned motifs around phosphorylation sites that would confer signaling specificity and fidelity (Tan et al., 2009; Zarrinpar et al., 2003), it is not so surprising that cancer mutations most often perturb this finely evolved system by generating weaker phosphorylation motifs.

Constitutive Activation and Inactivation of Kinases

As a final group of NAMs on protein kinases, we also analyzed the presence of mutations that would lead to the constitutive activation and inactivation of protein kinases (Figure 1).

Starting from the former case, we used the so-called phospho-mimicking effect of acidic mutations in close proximity to (just before, P−1, or after, P+1) activating phosphorylation sites on the activation segments of kinase domains (Davies et al., 2002) to identify in silico missense mutations that can result in a constitutively active kinase.

Taking the well-characterized case of BRAF V600E, a phospho-mimicking activating mutation, as a positive test case, we confirmed that ReKINect could identify this mutation in one of the ovarian cell lines (ES2) and predict it as kinase activation. We subsequently experimentally confirmed the hyper-phosphorylated state of the BRAF substrate, MEK by immuno-blot in the ES2 line (Figures 6A and 6B).

In addition to this well-known case, ReKINect predicted 23 other instances of potential constitutively activating kinase mutations (Table S4). Although some of these mutations fall nearby or on phosphorylation sites that have not yet been shown to regulate enzymatic activity, for a considerable fraction of them there is substantial evidence they could lead to kinase activation (Table S4). One exciting example of a predicted phospho-mimicking mutation was identified on the hematopoietic progenitor kinase 1 (HPK1), namely the mutant variant HPK1 A164D. Alanine 164 is immediately adjacent to the activating phosphorylation site T165 on the activation segment of HPK1, and mutation to Asp is predicted to confer constitutive activation of HPK1 and the likely engagement of its downstream JNK and NF-κB signaling (Arnold et al., 2005). Thus, the ReKINect predictions suggest a role for cellular stress response and potentially hematopoietic involvement in lung cancer, the cancer type in which this mutation was identified.

To model kinase inactivating mutations, we hypothesized that mutations that alter catalytically essential residues (e.g., residues mediating ATP binding, Mg^{2+} coordination or phosphotransfer, as defined in Zeqiraj and van Aalten, 2010 and Table S5) could lead to kinase inactivation. The high number of instances identified by ReKINect and detailed in Table S6 (427 unique kinase inactivation events) suggests that a large number of kinases become inactivated during cancer development. While it has previously been shown that inactivating mutations on kinases could lead to Peutz-Jeghers syndrome (Mehenni et al., 1998) or to pseudo-kinases throughout natural

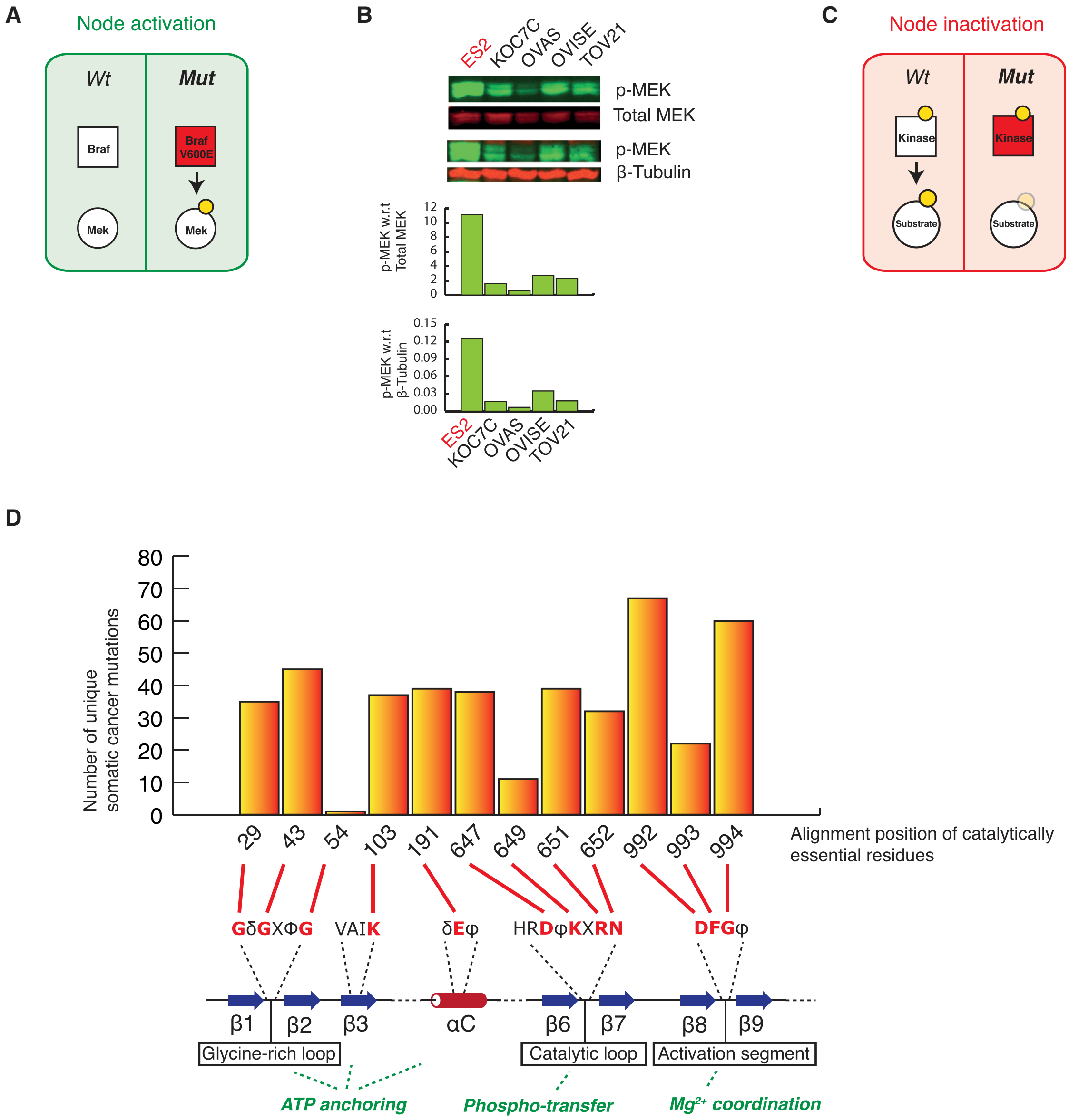

Figure 6. Constitutive Activation and Inactivation of Kinases by NAMs

(A) ReKINect identified ES2 cells as containing the constitutively activating BRAF V600E mutation.

(B) An immunoblot and associated quantification, illustrating the phosphorylation of BRAF substrate MEK in the mutant cell line ES2 (in red) compared to the wild-type cell lines (in black), using total MEK and β-tubulin for normalization.

(C) ReKINect identified several cancer mutations in catalytically essential residues of kinase domains.

(D) A quantification of all mutations from the global repository of cancer somatic mutations predicted to inactivate kinases and the catalytically essential positions they hit. Mutations leading to kinase domain catalytic inactivation are enriched (χ^2 test, $p = 1.69 \times 10^{-16}$) in cancer somatic mutations (with particular preference for the aspartate, D, and glycine, G, in the DFG motif).

evolution (Zeqiraj and van Aalten, 2010), our results indicate that kinase inactivation may hitherto have been largely under-appreciated in the interpretation of cancer genomes.

A closer inspection of these predicted inactivating mutations reveals a bias toward specific critical residues. In particular the first and third residues of the DFG motif (i.e., the glutamate and glycine, respectively) that defines the start of the activation segment, harbors approximately one-third of all inactivating mutations (Figures 6C and 6D). While mutations in other essential residues are likely to equally lead to kinase inactivation (see Table S5 for further information on the kinase catalytically essential residues included as part of ReKINect), our results suggest a significant preference for these two residues being mutated in the context of kinase inactivating mutations in cancer (χ^2 test, $p = 2.2 \times 10^{-16}$). Thus, these two positions of the DFG motif are predicted to constitute structural and

biochemical hotspots for NAMs leading to inactivation of protein kinases in cancer.

Overall, these results suggest that ReKINect is capable of predicting NAMs that constitutively activate or inactivate protein kinases and that, in addition to BRAF V600E, numerous other similar mutations are likely to exist that directly affect the catalytic activity of kinases in cancer signaling.

Functional Mutations in SH2 Domains and Global Phenotypic Impact of NAMs

The SH2 domain is of seminal importance to signaling fidelity, cellular organization, and function across Metazoan species and is often part of protein kinases and perturbed in human disease (Bibbins et al., 1993; Manning et al., 2002; Marengere et al., 1994; Pawson et al., 2001). Thus, we reasoned that the inclusion of the SH2 domain as part of ReKINect would enable us to make integrated predictions of higher accuracy and relevance from a signaling perspective. SH2 domains possess an essential Arg residue found within a highly conserved sequence motif (FLVRES) that makes direct contact with phosphoTyr residues in its binding partners (Bibbins et al., 1993). By incorporating this critical residue for the phospho-tyrosine binding function of SH2 domains into ReKINect, we could predict 20 distinct instances (including mutations on ABL, SYK, and GRB10) where cancer mutations disrupt a critical functional residue, thus impairing the ability of the mutant SH2 domains to bind their substrates (Table S7).

As with the mutations causing kinase downstream rewiring, by mapping cancer mutations onto the determinants of specificity of the SH2 domain identified by our algorithm KINspect (Creixell et al., 2015), ReKINect predicted 93 NAMs causing SH2 downstream rewiring (Table S8) by changing positions within the domain that show a high likelihood of playing a critical role in substrate specificity (specificity score higher than 0.9). The comparably lower number of inactivating and downstream rewiring mutations in SH2 domains compared with kinase domains, is attributable at least in part to the smaller number and size of SH2 domains in comparison with kinase domains (Figure S1).

Finally, to systematically explore the potential functional or phenotypic impact of the NAMs described above, we performed RNAi knockdown of kinase and SH2 domain containing proteins across the ovarian cancer cell lines. The effect of these perturbations on nuclear number was then determined using a regressor network model of protein-protein interactions and NAMs (Figure S3; Experimental Procedures). We found that if ReKINect classified NAMs were present in the network vicinity of the RNAi target gene a significant impact on the phenotypic response, either pro- or anti-proliferative, was observed ($p = 7.1 \times 10^{-13}$). These results would suggest that network attacking mutations, predicted by ReKINect, are not only biochemically functional but also lead to significant phenotypic changes in cancer cell models, on a global scale.

DISCUSSION

Given that protein kinases are one of the protein classes most frequently encoded by cancer genes (Futreal et al., 2004) and mutated in cancer (Figure S1) as well as a major molecular target of therapeutic drugs (Anastassiadis et al., 2011; Davis et al., 2011), it is essential to identify how phosphorylation-based signaling networks drive cancer. Thus, the number of distinct NAMs cataloged by ReKINect represents promising new leads for future studies. Serving as a systematic complement to previous efforts identifying the function that individual cancer mutations may play (Davies et al., 2002; Friend et al., 1986; Stehelin et al., 1976), ReKINect is designed to predict the underlying signaling mechanisms and perturbations caused by mutations in cancer, or other complex diseases, using first principles governing protein function from evolution, protein chemistry, and protein structure and architecture.

Evidence for NAMs and Signaling Trends in Cancer

Through integration of low and high-throughput computational and experimental technologies, we have discovered the existence of the NAMs described in Figure 1. Having analyzed the data generated here and in global genome sequencing efforts, we conclude that there is ample evidence supporting the hypothesis that all the different types of NAMs described do indeed occur in cancer.

In addition, our results also uncovered a variety of interesting signaling trends resulting from cancer mutations: first, our results demonstrate the existence of new molecular logic gates in cancer. The genesis of new phosphorylation sites by mutations as uncovered here illustrate how cancer cells can acquire novel and prominent signaling flows and altered information processing that may result in new phenotypic states to be reached.

We identified and experimentally confirmed three striking examples of cancer mutations directly leading to a catalytic specificity drift, PKD1 D665N, PKCγ D484N, and M501I. Downstream rewiring had until now been the most elusive type of NAMs, as reflected by the fact that only a single instance of this type of mutation, where a kinase is altered in specificity through mutation, RET M918T, had been reported in the literature (Borrello et al., 1995; Santoro et al., 1995; Songyang et al., 1995). The discovery of these three new NAMs, using a global yet selective and sensitive approach, would suggest that many more such events could exist in cancer.

Supporting this, we could pinpoint 16 additional cancer mutations that, given that they harbor identical amino acid substitutions to the ones we tested, are most likely to also encode downstream rewiring events. Next, when studying NAMs that would lead to downstream rewiring on a position that was recently confirmed to drive peptide specificity on the phosphoacceptor of phosphorylation sites (Chen et al., 2014), we could identify nine cancer mutations that are predicted to steer signaling toward phosphorylation of Thr, whereas no mutants were found in the opposite direction (Figure S4). Despite the fact that these numbers are not sufficiently high to enable robust statistics and that a large number of wild-type kinases originally encode Ser-directing residues in the DFG+1 position (thereby partially explaining the lack of Thr-to-Ser mutants), this bias suggests that specific cancers may harbor increased Thr-based signaling. Given that, due to its unique mutational and physicochemical properties, serine has been identified as a mutational hub (Creixell et al., 2012b) and thereby a likely result of cancer

mutations, we speculate that through such Ser-to-Thr signaling rewiring cancer cells might evolve less dependency on serine signaling.

Furthermore, if by affecting a large number of substrates, downstream rewiring NAMs are likely to have a broader impact at the network level, the fact that two of the downstream rewiring mutants described here lead to a less specific kinase and are thus likely to phosphorylate more substrates, highlights even further the potential impact of these NAMs. These results significantly extend our previous observations that less-specific kinases tend to be cancer mutation targets (Miller et al., 2008) and serve as a critical resource that we hope will start paving a new avenue on signaling specificity in cancer research.

Similar to the case of kinase downstream rewiring, relatively few mutations on SH2 domains had been reported to obliterate tyrosine-binding or shift their specificity (Marengere et al., 1994; Pawson et al., 2001), highlighting that this might also be a hitherto hidden and yet perhaps a fundamental signaling trend in cancer.

Finally, an over-representation of kinase-resignation upstream rewiring events suggests that cancer mutations most often lead to upstream rewiring by worsening existing optimal substrates rather than generating super-optimal new substrates for other upstream kinases. Given the amount of fine-tuning achieved over millions of years of evolution at the substrate level (Tan et al., 2009; Zarrinpar et al., 2003), it is perhaps to be expected that mutations in substrates will most often lead to poorer phosphorylation motifs.

Finally, our results suggest both the existence of previously unknown constitutively activating mutations in kinases as well as the presence of mutational hotspots on two specific positions leading to the inactivation of protein kinases, namely the Asp and Gly within the DFG motif at the beginning of the activation segment. It could be the aim of future studies to elucidate why these spots are preferred by cancer mutations when inactivating kinases.

Non-Recurrent yet Functional NAMs

While a large fraction of recurrent and/or conserved mutations can directly or indirectly be considered NAMs as they typically perturb signaling networks (as exemplified here with BRAF V600E) and they typically operate as functional driver mutations, in this study we have demonstrated that non-recurrent and non-conserved mutations also can be functional NAMs (Figure 7B). This may be most evident from the observation that downstream rewiring mutations can lead to a switch of specificity of a comparable magnitude to the specificity difference between two distinct kinases in the human kinome. Thus, despite the fact that previous studies of cancer mutations, including some on kinase domains, have disregarded non-recurrent variants as being non-functional passenger mutations (Greenman et al., 2007), our results suggest that many of these do indeed have a functional role. Still, pinning down the actual contribution of these less frequent yet functional mutations, or combinations thereof, and under which context they drive oncogenesis will require a concerted research effort by both the genomics and signaling communities. If we move from a perception of oncogenes and tumor-suppressors operating in isolation to drive oncogenesis, toward a new paradigm, where numerous mutations play a driving role under specific cellular contexts (e.g., when appearing in combination with other mutations) (Creixell et al., 2012a; Wu et al., 2010), it will be important to acknowledge that it is likely that several of these functional NAMs drive cancer in a concerted fashion.

As shown in Figure 7A, some of the NAMs we have identified here are likely to impose dramatic alterations in signaling networks, such as specificity switches that are analogous to introducing a new kinase and thus may play a driving role in oncogenesis.

The fact that there are multiple strategies in which the same signaling output can be achieved by distinct cancer mutations (as shown for instance by inactivating mutations in Figure 6D) and that we have identified overexpression of an instance of one of these functional NAMs, further supports the importance of such less frequent functional mutations (Figure 7B).

Perspectives

Our results suggest that signaling networks are both dynamically and structurally rewired in cancer cells to an extent far beyond what was previously anticipated. Such rewiring includes constitutive activation and inactivation of kinase and SH2 domains, upstream and downstream rewiring of phosphorylation-based signaling, and the extinction and genesis of phosphorylation sites. These findings will be critical for network medicine efforts where drug targets for complex diseases are defined at the network level and for the individual patient or tumor.

Here, we demonstrated six distinct NAMs as proof-of-principle and verified all the NAMs described in Figure 1A are present in cancer. Future expansions of the KINspect (Creixell et al., 2015) and ReKINect algorithms to include other protein domains, PTMs, and linear motifs, more complex genetic perturbations (such as copy-number variations or genomic re-arrangements leading to protein fusions) and the advancement of sequencing and MS technologies, will likely facilitate the discovery of many additional NAMs. Such advancements to link cancer genomic and proteomic data will become valuable resources for dealing with the intrinsic complexities of tumors (Weinberg, 2014; Yaffe, 2013).

In the last century, Schechter et al. (1984) and Ullrich et al. (1984) connected the discovery of the oncogene Her-2/neu to its hyperactivity in a fraction of breast cancers (Slamon et al., 1987) and the development of targeted therapies such as Trastuzumab (Carter et al., 1992). Others linked the discovery of the BCR-ABL fusion protein (Rowley, 1973) to CML leading to the development of Imatinib (Druker et al., 1996) and newer generation inhibitors. Similarly, we hope that ReKINect, and similar tools, can be utilized to close the cancer mutation interpretation gap. Boosting genomic interpretation capacity should ideally parallel the rate at which next generation technologies identify new mutations in order to help meet the bench-to-bedside challenge (Figure 7C), assist clinicians in making better treatment decisions for those patients carrying infrequent yet functional cancer mutations and facilitate the development of novel “magic bullets” (Strebhardt and Ullrich, 2008) and precision medicines (Creixell et al., 2012a).

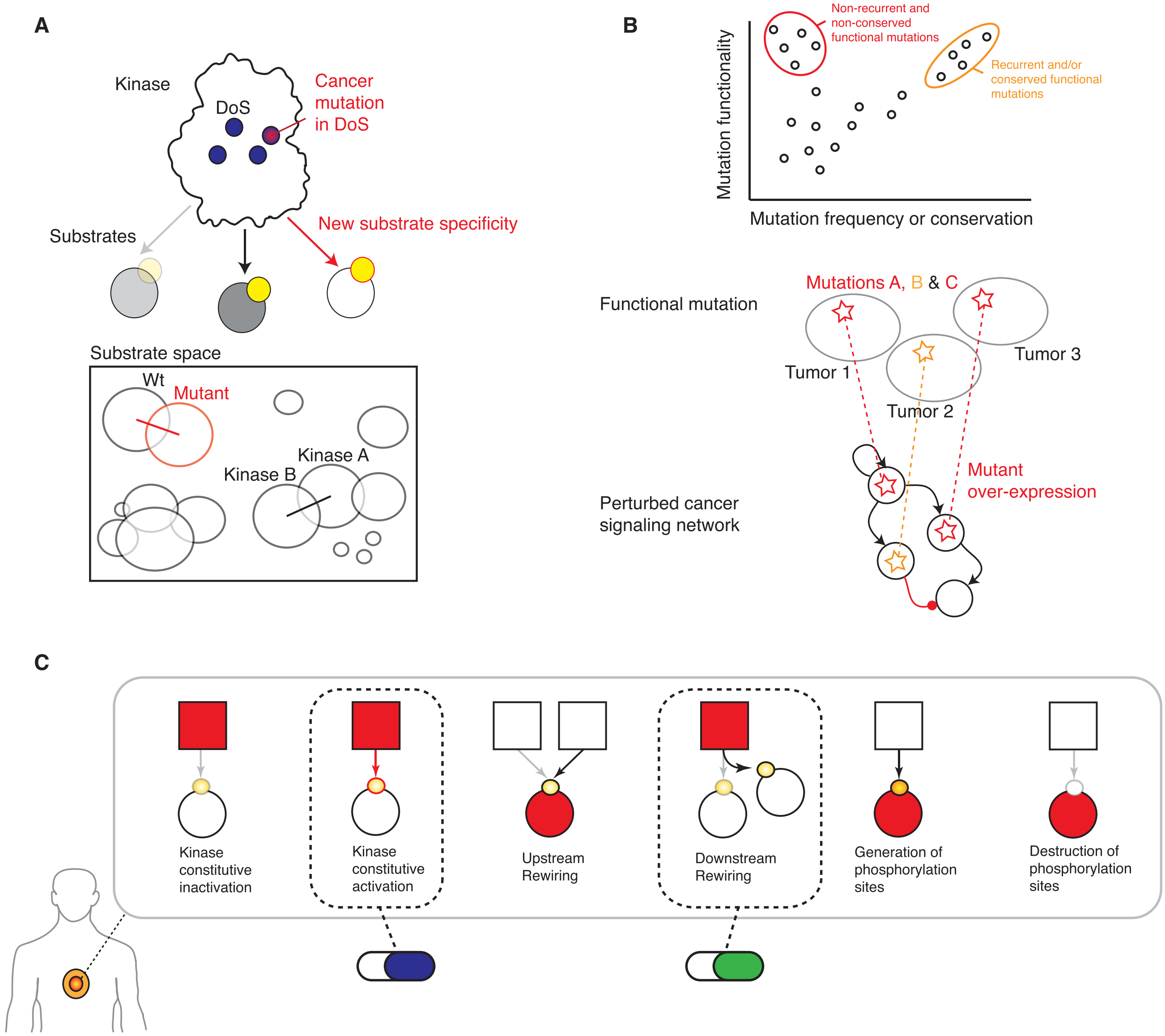

Figure 7. Evidence and Model for Functional Mutations and Tumor-Specific Network Medicine

(A) The functional mutations found in this study are clear examples of single amino acid mutations that can severely perturb signaling networks.

(B) Our study shows how non-recurrent cancer mutations on non-conserved residues can be functionally important and that functional recurrent (orange) and non-recurrent (red) NAMs can converge at the signaling network level. We also identified a case where a functional mutation in a low-abundant protein is accompanied by its overexpression.

(C) The deployment of tools like ReKINect should enable the proposition of more refined signaling mechanisms underlying cellular cancer phenotypes and identification of driver and therapeutically relevant mutations.

EXPERIMENTAL PROCEDURES

Building Comprehensive Sets of Sequences: Kinome, SH2ome, and Phosphorylation Sites

We built comprehensive sets of sequences covering all human kinase proteins (Manning et al., 2002), 120 SH2 domains (Liu et al., 2006), and a broad set of known human phosphorylation sites (Hornbeck et al., 2004). With these sets, we performed domain-centered sequence alignments using ClustalW and Omega (Sievers et al., 2011) followed by subsequent manual refinement. These alignments were then deployed by identifying functional residues on them and mapping these residues back to the wild-type version of the mutant sequences analyzed with ReKINect. Similarly, phosphorylation site peptides were matched to the wild-type variants of all mutations, so that the distance between each mutation and its closest phosphorylation sites could be determined.

Collecting a Global Repository of Somatic Cancer Mutations

We compiled a global set of publicly available somatic cancer mutations from COSMIC v67 (Forbes et al., 2011) and generated the FASTA files required by ReKINect containing both the wild-type and mutant versions of all coding missense variants, using purpose-made Python scripts and ENSEMBL's VEP resource (Flicek et al., 2014).

Computing Minimum Distance to Substrate from PDB Files

Minimum distances to substrates were computed as described in the accompanying article (Creixell et al., 2015) and further detailed in the Supplemental Experimental Procedures.

Protein Kinase Specificity Assays

Kinases and mutants were expressed by transient transfection of encoding plasmids in HEK293T cells, purified by FLAG affinity purification, and PSPL experiments were performed as described (Mok et al., 2010). Further details can be found in the Supplemental Experimental Procedures.

Further details about the maintenance of cell lines, preparation of sequencing, mass spectrometry, and RNAi screening samples and their computational analysis can similarly be found in the Supplemental Experimental Procedures.

SUPPLEMENTAL INFORMATION

Supplemental Information includes Supplemental Experimental Procedures, five figures, eight tables, and six data files and can be found with this article online at http://dx.doi.org/10.1016/j.cell.2015.08.056.

AUTHOR CONTRIBUTIONS

P.C. and R.L. conceived the project. P.C., E.M.S., C.D.S., J.L., C.J.M., H.J.L., L.P., T.R.C., N.Z., and B.B. performed wet-lab experiments. P.C., E.M.S., A.P., A.W., and J.F.-B. performed computational experiments. P.C. generated the lists of NAMs assisted by A.P. P.C. and B.E.T. prioritized the list of NAMs likely to drive downstream rewiring. H.I. provided cell lines. All authors analyzed different parts of the data generated. P.C. and R.L. wrote the article with help from the other authors.

ACKNOWLEDGMENTS

This article is dedicated to the memory of late Tony Pawson, whose groundbreaking research represented a fundamental foundation to this work. We thank T. Gibson for technical assistance with sequence alignments, S. Knapp's group and the Structural Genomics Consortium for providing plasmids, G. Manning for sharing unpublished data, and members of the R.L. laboratory for critical reading of this manuscript, particularly C. Santini. We are further indebted to J. Kim for assistance with NetworKIN, NetPhorest, and KinomeXplorer and X. Robin for assisting in developing and maintaining the ReKINect website. We are also indebted to several members of the Pawson laboratory, especially A. Pasculescu for his assistance in collecting phosphorylation site information, G. Gish for discussions on potentially essential SH2 residues, and B. Larsen for his suggestions on MS spectra annotation. Similarly, we thank members of the Yaffe laboratory for discussions on critical parts of this manuscript. This work was funded by The Lundbeck Foundation and the European Research Council under the European Union's Seventh Framework Programme (FP/2007-2013)/ERC grant (KINOMEDRIFT) and was conducted using the VKR funded Instrument Center for Systems Proteomics (VKR 022758). J.T.E. and L.P. are supported by a Hallas Møller Stipend from the Novo Nordisk Foundation. Work in the B.E.T. laboratory was partly supported by NIH grant R01 GM104047, and C.J.M. was supported by NIH training grant T32 GM007324. P.C. is currently funded by a Ludwig Fund Postdoctoral Fellowship. The algorithms and software developed in this work will be released under the Creative Commons licensing schemes at the websites http://KinomeXplorer.science, http://ReKINect.science, and http://KINspect.science. For further information see also http://lindinglab.science.

Received: August 25, 2014
Revised: April 9, 2015
Accepted: August 12, 2015
Published: September 17, 2015

REFERENCES

Alexander, J., Lim, D., Joughin, B.A., Hegemann, B., Hutchins, J.R.A., Ehrenberger, T., Ivins, F., Sessa, F., Hudecz, O., Nigg, E.A., et al. (2011). Spatial exclusivity combined with positive and negative selection of phosphorylation motifs is the basis for context-dependent mitotic signaling. Sci. Signal. *4*, ra42.

Anastassiadis, T., Deacon, S.W., Devarajan, K., Ma, H., and Peterson, J.R. (2011). Comprehensive assay of kinase catalytic activity reveals features of kinase inhibitor selectivity. Nat. Biotechnol. *29*, 1039–1045.

Andreadi, C., Cheung, L.K., Giblett, S., Patel, B., Jin, H., Mercer, K., Kamata, T., Lee, P., Williams, A., McMahon, M., et al. (2012). The intermediate-activity (L597V)BRAF mutant acts as an epistatic modifier of oncogenic RAS by enhancing signaling through the RAF/MEK/ERK pathway. Genes Dev. *26*, 1945–1958.

Antal, C.E., Hudson, A.M., Kang, E., Zanca, C., Wirth, C., Stephenson, N.L., Trotter, E.W., Gallegos, L.L., Miller, C.J., Furnari, F.B., et al. (2015). Cancer-associated protein kinase C mutations reveal kinase's role as tumor suppressor. Cell *160*, 489–502.

Arnold, R., Patzak, I.M., Neuhaus, B., Vancauwenbergh, S., Veillette, A., Van Lint, J., and Kiefer, F. (2005). Activation of hematopoietic progenitor kinase 1 involves relocation, autophosphorylation, and transphosphorylation by protein kinase D1. Mol. Cell. Biol. *25*, 2364–2383.

Bibbins, K.B., Boeuf, H., and Varmus, H.E. (1993). Binding of the Src SH2 domain to phosphopeptides is determined by residues in both the SH2 domain and the phosphopeptides. Mol. Cell. Biol. *13*, 7278–7287.

Borrello, M.G., Smith, D.P., Pasini, B., Bongarzone, I., Greco, A., Lorenzo, M.J., Arighi, E., Miranda, C., Eng, C., Alberti, L., et al. (1995). RET activation by germline MEN2A and MEN2B mutations. Oncogene *11*, 2419–2427.

Brinkworth, R.I., Breinl, R.A., and Kobe, B. (2003). Structural basis and prediction of substrate specificity in protein serine/threonine kinases. Proc. Natl. Acad. Sci. USA *100*, 74–79.

Carter, P., Presta, L., Gorman, C.M., Ridgway, J.B., Henner, D., Wong, W.L., Rowland, A.M., Kotts, C., Carver, M.E., and Shepard, H.M. (1992). Humanization of an anti-p185HER2 antibody for human cancer therapy. Proc. Natl. Acad. Sci. USA *89*, 4285–4289.

Chen, C., Ha, B.H., Thévenin, A.F., Lou, H.J., Zhang, R., Yip, K.Y., Peterson, J.R., Gerstein, M., Kim, P.M., Filippakopoulos, P., et al. (2014). Identification of a major determinant for serine-threonine kinase phosphoacceptor specificity. Mol. Cell *53*, 140–147.

Creixell, P., Schoof, E.M., Erler, J.T., and Linding, R. (2012a). Navigating cancer network attractors for tumor-specific therapy. Nat. Biotechnol. *30*, 842–848.

Creixell, P., Schoof, E.M., Tan, C.S.H., and Linding, R. (2012b). Mutational properties of amino acid residues: implications for evolvability of phosphorylatable residues. Philos. Trans. R. Soc. Lond. B Biol. Sci. *367*, 2584–2593.

Creixell, P., Palmeri, A., Miller, C.J., Lou, H.J., Santini, C.C., Nielsen, M., Turk, B.E., and Lining, R. (2015). Unmasking determiniants of specifity in the human kinome. Cell *163*, this issue, 187–201.

Dai, C., Whitesell, L., Rogers, A.B., and Lindquist, S. (2007). Heat shock factor 1 is a powerful multifaceted modifier of carcinogenesis. Cell *130*, 1005–1018.

Davies, H., Bignell, G.R., Cox, C., Stephens, P., Edkins, S., Clegg, S., Teague, J., Woffendin, H., Garnett, M.J., Bottomley, W., et al. (2002). Mutations of the BRAF gene in human cancer. Nature *417*, 949–954.

Davis, M.I., Hunt, J.P., Herrgard, S., Ciceri, P., Wodicka, L.M., Pallares, G., Hocker, M., Treiber, D.K., and Zarrinkar, P.P. (2011). Comprehensive analysis of kinase inhibitor selectivity. Nat. Biotechnol. *29*, 1046–1051.

Druker, B.J., Tamura, S., Buchdunger, E., Ohno, S., Segal, G.M., Fanning, S., Zimmermann, J., and Lydon, N.B. (1996). Effects of a selective inhibitor of the Abl tyrosine kinase on the growth of Bcr-Abl positive cells. Nat. Med. *2*, 561–566.

Flicek, P., Amode, M.R., Barrell, D., Beal, K., Billis, K., Brent, S., Carvalho-Silva, D., Clapham, P., Coates, G., Fitzgerald, S., et al. (2014). Ensembl 2014. Nucleic Acids Res. *42*, D749–D755.

Forbes, S.A., Bindal, N., Bamford, S., Cole, C., Kok, C.Y., Beare, D., Jia, M., Shepherd, R., Leung, K., Menzies, A., et al. (2011). COSMIC: mining complete cancer genomes in the Catalogue of Somatic Mutations in Cancer. Nucleic Acids Res. *39*, D945–D950.

Friend, S.H., Bernards, R., Rogelj, S., Weinberg, R.A., Rapaport, J.M., Albert, D.M., and Dryja, T.P. (1986). A human DNA segment with properties of the gene that predisposes to retinoblastoma and osteosarcoma. Nature *323*, 643–646.

Futreal, P.A., Coin, L., Marshall, M., Down, T., Hubbard, T., Wooster, R., Rahman, N., and Stratton, M.R. (2004). A census of human cancer genes. Nat. Rev. Cancer *4*, 177–183.

Gao, J., Aksoy, B.A., Dogrusoz, U., Dresdner, G., Gross, B., Sumer, S.O., Sun, Y., Jacobsen, A., Sinha, R., Larsson, E., et al. (2013). Integrative analysis of complex cancer genomics and clinical profiles using the cBioPortal. Sci. Signal. *6*, pl1.

Geiger, T., Wisniewski, J.R., Cox, J., Zanivan, S., Kruger, M., Ishihama, Y., and Mann, M. (2011). Use of stable isotope labeling by amino acids in cell culture as a spike-in standard in quantitative proteomics. Nat. Protoc. *6*, 147–157.

Greenman, C., Stephens, P., Smith, R., Dalgliesh, G.L., Hunter, C., Bignell, G., Davies, H., Teague, J., Butler, A., Stevens, C., et al. (2007). Patterns of somatic mutation in human cancer genomes. Nature *446*, 153–158.

Hanahan, D., and Weinberg, R.A. (2000). The hallmarks of cancer. Cell *100*, 57–70.

Hassan, S., Biswas, M.H.U., Zhang, C., Du, C., and Balaji, K.C. (2009). Heat shock protein 27 mediates repression of androgen receptor function by protein kinase D1 in prostate cancer cells. Oncogene *28*, 4386–4396.

Hornbeck, P.V., Chabra, I., Kornhauser, J.M., Skrzypek, E., and Zhang, B. (2004). PhosphoSite: a bioinformatics resource dedicated to physiological protein phosphorylation. Proteomics *4*, 1551–1561.

Hutti, J.E., Jarrell, E.T., Chang, J.D., Abbott, D.W., Storz, P., Toker, A., Cantley, L.C., and Turk, B.E. (2004). A rapid method for determining protein kinase phosphorylation specificity. Nat. Methods *1*, 27–29.

Kan, Z., Jaiswal, B.S., Stinson, J., Janakiraman, V., Bhatt, D., Stern, H.M., Yue, P., Haverty, P.M., Bourgon, R., Zheng, J., et al. (2010). Diverse somatic mutation patterns and pathway alterations in human cancers. Nature *466*, 869–873.

Kramer, K.L., Barnette, J.E., and Yost, H.J. (2002). PKCgamma regulates syndecan-2 inside-out signaling during xenopus left-right development. Cell *111*, 981–990.

Lindberg, J., Mills, I.G., Klevebring, D., Liu, W., Neiman, M., Xu, J., Wikström, P., Wiklund, P., Wiklund, F., Egevad, L., and Grönberg, H. (2013). The mitochondrial and autosomal mutation landscapes of prostate cancer. Eur. Urol. *63*, 702–708.

Linding, R., Jensen, L.J., Ostheimer, G.J., van Vugt, M.A.T.M., Jørgensen, C., Miron, I.M., Diella, F., Colwill, K., Taylor, L., Elder, K., et al. (2007). Systematic discovery of in vivo phosphorylation networks. Cell *129*, 1415–1426.

Liu, B.A., Jablonowski, K., Raina, M., Arcé, M., Pawson, T., and Nash, P.D. (2006). The human and mouse complement of SH2 domain proteins-establishing the boundaries of phosphotyrosine signaling. Mol. Cell *22*, 851–868.

Manning, G., Whyte, D.B., Martinez, R., Hunter, T., and Sudarsanam, S. (2002). The protein kinase complement of the human genome. Science *298*, 1912–1934.

Marengere, L.E., Songyang, Z., Gish, G.D., Schaller, M.D., Parsons, J.T., Stern, M.J., Cantley, L.C., and Pawson, T. (1994). SH2 domain specificity and activity modified by a single residue. Nature *369*, 502–505.

Mazzoni, E., Adam, A., Bal de Kier Joffe, E., and Aguirre-Ghiso, J.A. (2003). Immortalized mammary epithelial cells overexpressing protein kinase C gamma acquire a malignant phenotype and become tumorigenic in vivo. Mol. Cancer Res. *1*, 776–787.

Mehenni, H., Gehrig, C., Nezu, J., Oku, A., Shimane, M., Rossier, C., Guex, N., Blouin, J.-L., Scott, H.S., and Antonarakis, S.E. (1998). Loss of LKB1 kinase activity in Peutz-Jeghers syndrome, and evidence for allelic and locus heterogeneity. Am. J. Hum. Genet. *63*, 1641–1650.

Miller, M.L., Jensen, L.J., Diella, F., Jørgensen, C., Tinti, M., Li, L., Hsiung, M., Parker, S.A., Bordeaux, J., Sicheritz-Ponten, T., et al. (2008). Linear motif atlas for phosphorylation-dependent signaling. Sci. Signal. *1*, ra2.

Mok, J., Kim, P.M., Lam, H.Y.K., Piccirillo, S., Zhou, X., Jeschke, G.R., Sheridan, D.L., Parker, S.A., Desai, V., Jwa, M., et al. (2010). Deciphering protein kinase specificity through large-scale analysis of yeast phosphorylation site motifs. Sci. Signal. *3*, ra12.

Pawson, T., Gish, G.D., and Nash, P. (2001). SH2 domains, interaction modules and cellular wiring. Trends Cell Biol. *11*, 504–511.

Rowley, J.D. (1973). Letter: A new consistent chromosomal abnormality in chronic myelogenous leukaemia identified by quinacrine fluorescence and Giemsa staining. Nature *243*, 290–293.

Santoro, M., Carlomagno, F., Romano, A., Bottaro, D.P., Dathan, N.A., Grieco, M., Fusco, A., Vecchio, G., Matoskova, B., Kraus, M.H., et al. (1995). Activation of RET as a dominant transforming gene by germline mutations of MEN2A and MEN2B. Science *267*, 381–383.

Schechter, A.L., Stern, D.F., Vaidyanathan, L., Decker, S.J., Drebin, J.A., Greene, M.I., and Weinberg, R.A. (1984). The neu oncogene: an erb-B-related gene encoding a 185,000-Mr tumour antigen. Nature *312*, 513–516.

Sievers, F., Wilm, A., Dineen, D., Gibson, T.J., Karplus, K., Li, W., Lopez, R., McWilliam, H., Remmert, M., Söding, J., et al. (2011). Fast, scalable generation of high-quality protein multiple sequence alignments using Clustal Omega. Mol. Syst. Biol. *7*, 539.

Slamon, D.J., Clark, G.M., Wong, S.G., Levin, W.J., Ullrich, A., and McGuire, W.L. (1987). Human breast cancer: correlation of relapse and survival with amplification of the HER-2/neu oncogene. Science *235*, 177–182.

Songyang, Z., Carraway, K.L., 3rd, Eck, M.J., Harrison, S.C., Feldman, R.A., Mohammadi, M., Schlessinger, J., Hubbard, S.R., Smith, D.P., Eng, C., et al. (1995). Catalytic specificity of protein-tyrosine kinases is critical for selective signalling. Nature *373*, 536–539.

Stehelin, D., Varmus, H.E., Bishop, J.M., and Vogt, P.K. (1976). DNA related to the transforming gene(s) of avian sarcoma viruses is present in normal avian DNA. Nature *260*, 170–173.

Strebhardt, K., and Ullrich, A. (2008). Paul Ehrlich's magic bullet concept: 100 years of progress. Nat. Rev. Cancer *8*, 473–480.

Sundram, V., Chauhan, S.C., and Jaggi, M. (2011). Emerging roles of protein kinase D1 in cancer. Mol. Cancer Res. *9*, 985–996.

Tan, C.S.H., Pasculescu, A., Lim, W.A., Pawson, T., Bader, G.D., and Linding, R. (2009). Positive selection of tyrosine loss in metazoan evolution. Science *325*, 1686–1688.

Thomsen, M.C.F., and Nielsen, M. (2012). Seq2Logo: a method for construction and visualization of amino acid binding motifs and sequence profiles including sequence weighting, pseudo counts and two-sided representation of amino acid enrichment and depletion. Nucleic Acids Res. *40*, W281–W287.

Ullrich, A., Coussens, L., Hayflick, J.S., Dull, T.J., Gray, A., Tam, A.W., Lee, J., Yarden, Y., Libermann, T.A., Schlessinger, J., et al. (1984). Human epidermal growth factor receptor cDNA sequence and aberrant expression of the amplified gene in A431 epidermoid carcinoma cells. Nature *309*, 418–425.

Weinberg, R.A. (2014). Coming full circle-from endless complexity to simplicity and back again. Cell *157*, 267–271.

Wu, M., Pastor-Pareja, J.C., and Xu, T. (2010). Interaction between Ras(V12) and scribbled clones induces tumour growth and invasion. Nature *463*, 545–548.

Yaffe, M.B. (2013). The scientific drunk and the lamppost: massive sequencing efforts in cancer discovery and treatment. Sci. Signal. *6*, pe13.

Yang, J., Song, X., Chen, Y., Lu, X., Fu, Y., and Luo, Y. (2014). PLCγ1-PKCγ signaling-mediated Hsp90α plasma membrane translocation facilitates tumor metastasis. Traffic *15*, 861–878.

Zarrinpar, A., Park, S.H., and Lim, W.A. (2003). Optimization of specificity in a cellular protein interaction network by negative selection. Nature *426*, 676–680.

Zeqiraj, E., and van Aalten, D.M. (2010). Pseudokinases-remnants of evolution or key allosteric regulators? Curr. Opin. Struct. Biol. *20*, 772–781.

Supplemental Figures

A

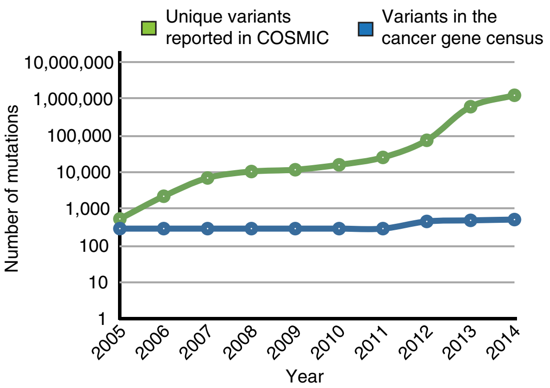

B

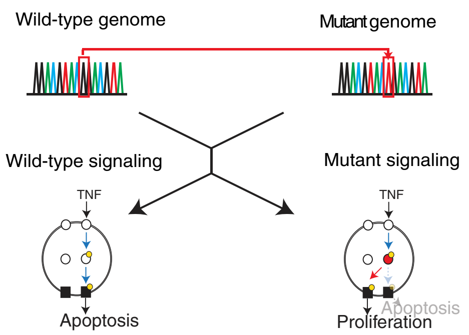

C

Cell Line	Origin	Classification
ES2	Primary tumor	Atypical non-serous ovarian carcinoma
KOC7C	Pleural effusion	Clear cell ovarian carcinoma
OVAS	Ascites	Clear cell ovarian carcinoma
OVISE	Metastasis	Atypical non-serous ovarian carcinoma
TOV21	Primary tumor	Clear cell ovarian carcinoma

D

	NGS Data		Phospho-Proteomic Data				Proteomic Data
Cell Line	Number of mutations	Number of mutated proteins	Number of phosphorylation sites	Number of confidently-localized phosphorylation sites*	Number of phosphorylated proteins	Number of phosphorylated proteins with at least 1 confidently-localized* phosphorylation site	Number of confidently identified** and quantified proteins
ES2	8707	5273	6680	5885	2306	2234	4539
KOC7C	9735	5965	3578	3307	1607	1562	3842
OVAS	9307	5606	6569	5755	2419	2333	4052
TOV21	9014	5390	5141	4643	2019	1963	3945
OVISE	8163	4937	6134	5329	2084	2001	4400

* MS localization score >= 0.75

** >1 peptide with MaxQuant Score >= 42

E

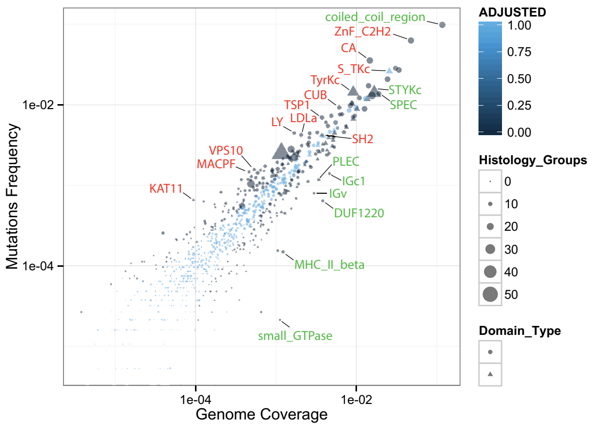

F

FUNCTIONAL RESIDUE TYPE	ENRICHMENT ODDS RATIO	P-VALUE	CORRECTED P-VALUE	FUCTIONAL_MUT	NONFUNCTIONAL_MUT	FUCTIONAL_NONMUT	NONFUNCTIONAL_NONMUT
KIN_ESSENTIAL	1.59	5.63E-17	1.69E-16	402	641428	4658	11815899
KIN_DoS	1.33	2.71E-30	1.08E-29	1888	639942	26192	11794365
SH2_ESSENTIAL	3.53	1.74E-05	3.47E-05	18	641812	94	11820463
SH2_DoS	1.18	0.09	9.45E-02	113	641717	1765	11818792
PSITES	1.12	1.82E-157	9.08E-157	66846	574984	1110959	10709598

(legend on next page)

Figure S1. Interpreting Functional Cancer Somatic Mutations in Repositories of Cancer Genome Data and Cell Lines, Related to Figures 1 and 2

(A) The gap between the number of unique cancer somatic mutations reported by global sequencing efforts and the ones for which the community has been able to attribute a driving role in cancer (list of genes in the cancer gene census [Forbes et al., 2011]) has been growing drastically in the last years.

(B) A more comprehensive understanding of signaling networks and how mutations perturb them would help close the interpretation gap described in (A).

(C and D) Data summary of the provenance (C) and different experimental observations (D) concerning mutations, phosphorylation sites, and proteins found using exome sequencing (NGS) and (phospho)proteomics-based mass spectrometry. Stringent filters were applied to ensure data quality, including 95% of sequence space covered by 10X sequencing reads for the NGS data, standard filters applied in subsequent steps (see Experimental Procedures), and high MaxQuant and localization scores for the MS data (see Experimental Procedures).

(E) Using the global repository of somatic cancer mutations, we quantified the enrichment of mutations in functional residues covered by ReKINect, and to what extent different protein domains are affected by somatic missense mutations. As one would expect, and can be observed in (E), the mutation frequency generally depends on the fraction of the genome that a given domain covers (genome coverage), as shown in the scatter plot. However, several signaling (triangles) and non-signaling (circles) domains harbor many more mutations than it would be expected by random chance or genome coverage alone (darker blue denoting lower P-value) and are mutated in a wider range of cancers (data point size). These include signaling domains like serine-threonine kinase domains, S_TKc, tyrosine kinase domains, TyrKc, and SH2 domains.

(F) The results on (B) show the enrichment in cancer mutations on specific residues, calculated as the fraction of functional residues mutated and not mutated with respect to the fraction of the proteome covered by functional and non-functional residues. Odds-ratios and P-values were computed using a Fisher's Exact Test with Multiple-Test Correction.

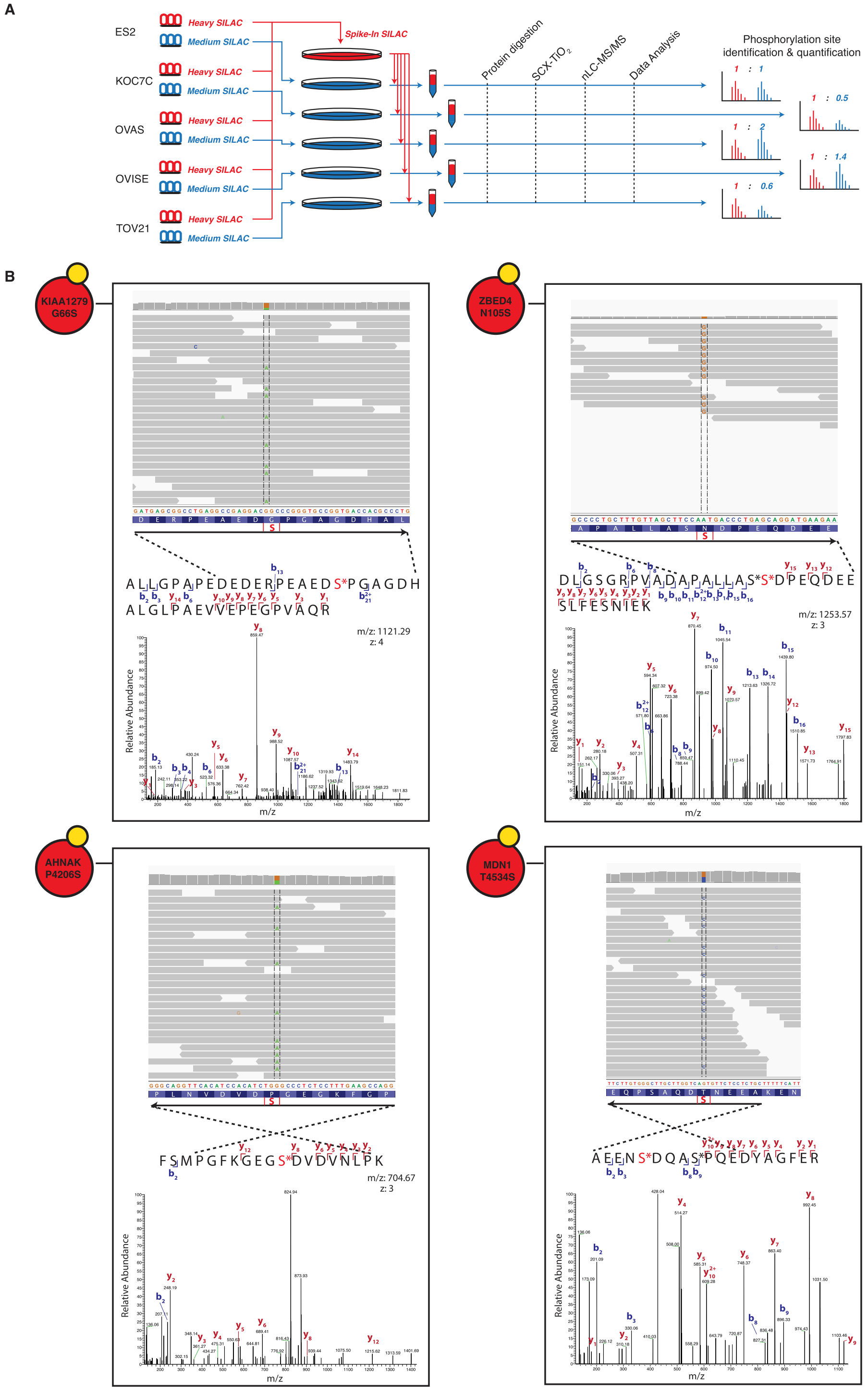

Figure S2. Genesis of Phosphorylation Sites by Cancer Mutations Experimentally Confirmed by Mass Spectrometry, Related to Figure 3

(A) Samples are processed following Spike-In SILAC standard procedures, where a mix of all the samples, in our case the five ovarian cancer cell lines, is used as an internal reference, so that peptides from different samples can be compared (Geiger et al., 2011; Monetti et al., 2011).

(B) Additional examples of NGS and MS-annotated NAMs generating phosphorylation sites, including KIAA1279 G66S, ZBED4 N105S, AHNAK P4206S and MDN1 T4534S, showing the mapping of NGS and MS data, where the mutation toward phosphorylatable residues are confirmed as phosphorylation sites by the MS data using genome-specific searches.

A

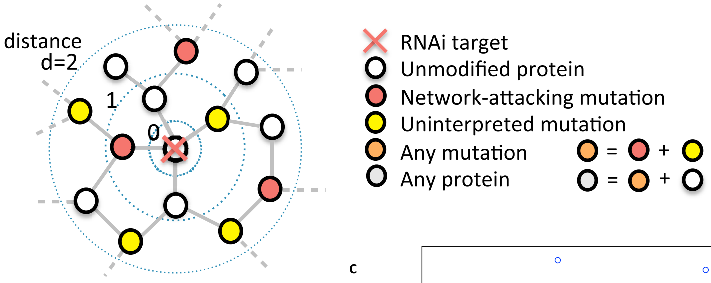

B

	Estimate	P-value
**NAM-Model**		
d=2	-0.4 ± 0.1	$3.3x10^{-9}$
d=2	-0.4 ± 0.1	$1.1x10^{-13}$
**Classified-model**		
d=1	-0.4 ± 0.1	$6.9x10^{-3}$
d=2	-0.4 ± 0.1	$2.8x10^{-12}$
d=2	-0.4 ± 0.1	$<2x10^{-16}$

C

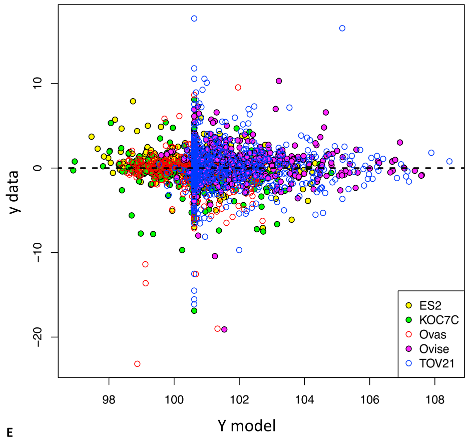

D E F

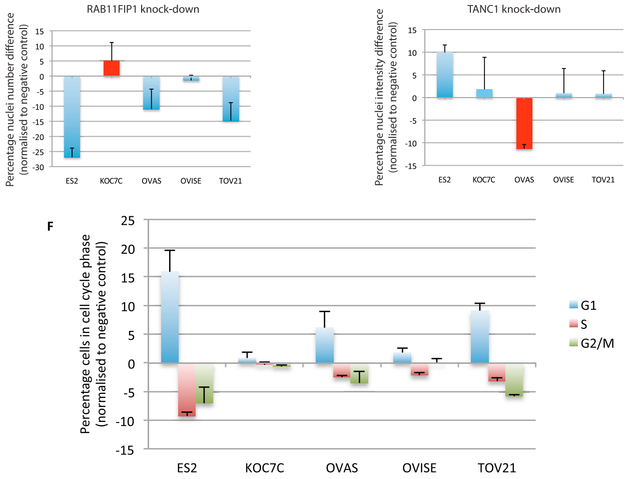

Figure S3. Global Analysis of the Effect of Network Attacking Mutations on Cell Phenotypes, Related to Figure 3

(A) The effects of network topology, the presence of network attacking mutations and the classification of those mutations on the phenotypic impact of RNAi knockdown of kinase and SH2 domain containing genes were analyzed using a regression model (see Supplemental Experimental Procedures).

(B) Regression variables determining model performance. For the NAM-model RNAi targets with NAMs in their close vicinity (d = 2) are more likely to cause phenotypic changes when knocked down. This likelihood is increased further if the classification of NAM is taken into account (Classified-model d = 1 and d = 2). In both models, the more proteins there are in the network vicinity of the RNAi target the less likely it is a phenotype will be observed (Any protein, d = 2) (see Supplemental Experimental Procedures).

(C) The final regression result for the NAM model. Y model illustrates the regression model prediction expressed in terms of the number of nuclei, normalized to negative control, y data show the deviation from the model prediction expressed in terms of the experimental SD (see Supplemental Experimental Procedures).

(D) RAB11FIP1 knockdown leads to a reduction in proliferation in all the $RAB11FIP1^{wt}$ cell lines (ES2, OVAS, OVISE and TOV21). This response is lost in the cell line where a mutation has led to the extinction of a phosphorylation site on RAB11FIP1 (RAB11FIP1 T281M), KOC7C. Data shown is mean + SD from quadruplicate biological repeats.

(E) TANC1 knockdown leads to a reduction in nuclear intensity in the OVAS cell line, in which a new phosphorylation site has appeared as a result of a mutation (TANC1 N251S). $TANC1^{wt}$ cell lines (ES2, KOC7C, OVISE and TOV21) showed no decrease in intensity. Data shown is mean + SD from quadruplicate biological repeats.

(F) The RAB11FIP1 mutated cell line KOC7C was the only cell line not to show a significant phenotype in FACS analysis of cell-cycle dynamics. Data shown is mean + SD from triplicate biological repeats.

A

Kinase name (including group & family)	*Cancer mutation*
AGC-PKC-PKCa-PKCg	M501I
Other-Haspin--Haspin	L669I
TK-DDR--DDR1	M793I
TK-Tec--ITK	M503V
TK-Trk--TRKA	M671T
TKL-IRAK--IRAK3	M314I
TKL-IRAK--IRAK3	M314V
TKL-IRAK--IRAK3	M314T
TKL-RAF-RAF-BRAF	L597V

B

Kinase name (including group & family)	*Cancer mutation*
AGC-PKC-PKCa-PKCb	D427N
CAMK-PKD--PKD1	D665N
CAMK-TSSK-TSSK1	D97N
CK1-TTBK--TTBK1	D116N
CMGC-CDK-CDK11-CDK11b	D507N
CMGC-CDK-CDK8-CDK8	D103N
CMGC-CDK-PFTAIRE-PFTAIRE1	D198N
TK-PVR-PDGFR-PDGFRa	D681N
TK-TK-Unique--STYK1	D201N

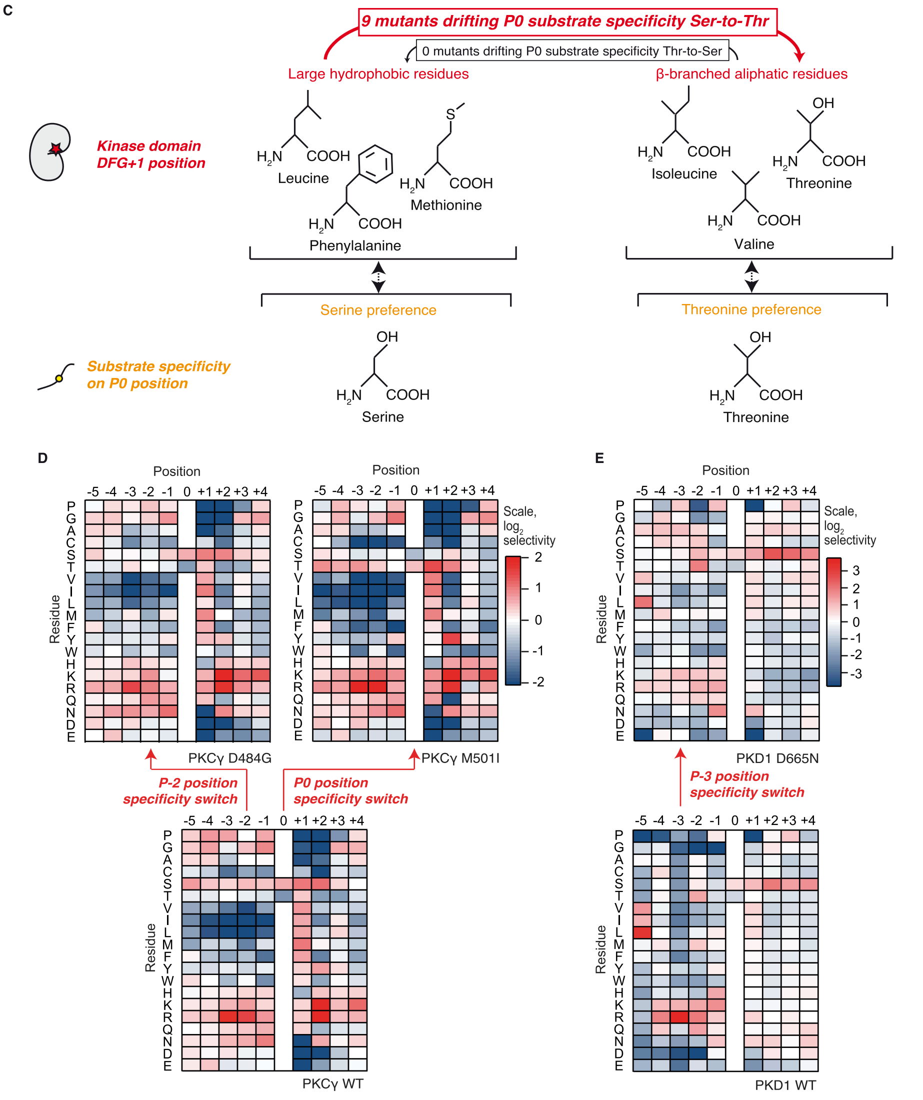

Figure S4. High-Confidence Candidate NAMs Driving Downstream Rewiring and Downstream Rewiring Experimental Validation, Related to Figure 4

(A) Cancer mutants on position DFG+1 most likely causing downstream rewiring. Further focusing on alignment position DFG+1 reveals several additional cancer mutations with hydrophobic-to-β-branched aliphatic residues.

(B) Cancer mutants on position αD1 most likely causing downstream rewiring. Further focusing on alignment position αD1 reveals several additional cancer mutations with D-to-N substitutions.

(C) The enrichment of mutations in DFG+1 favoring Ser-to-Thr specificity switches (with nine mutants following this pattern and none in the opposite direction, as shown in the bottom figure) will most likely lead to a specificity drift from Serine toward Threonine phosphorylation preference.

(D) Full PSSMs for the downstream rewiring cancer mutation in PKCγ driving changes in kinase substrate specificity. Two cancer mutations in PKCγ, D484G and M501I, switch specificity by perturbing the residue preference on the substrate position P-2 and P0 (i.e., the phospho-acceptor site) respectively.

(E) Full PSSMs for the downstream rewiring cancer mutation in PKD1 driving changes in kinase substrate specificity. A cancer mutation in PKD1, D665N, causes a drift in substrate specificity on P-3 position.

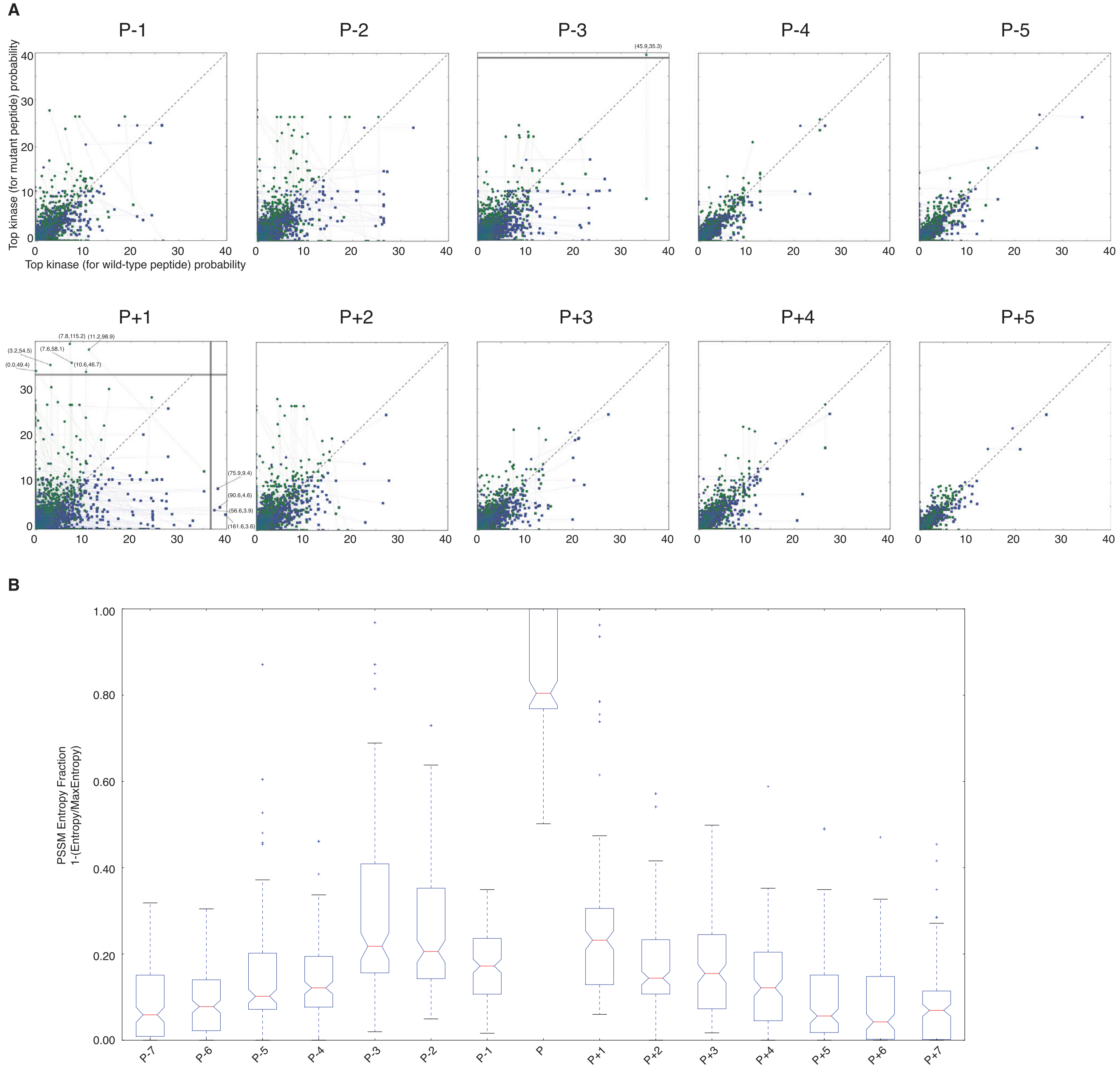

Figure S5. Upstream Rewiring Graphs Using NetworKIN and Information Content in Various Phosphorylation Substrate Positions, Related to Figure 5

(A) As an extension to the graphs shown in Figure 5, here we show similar rewiring graphs computed using NetworKIN (Linding et al., 2007) instead of NetPhorest (Miller et al., 2008), and therefore including contextual information for improved accuracy. Please note that the extreme values in P-3 and P+1 graphs were added for completeness, but due to their outlier status are out of scale (values added and their numerical values can be seen in the figure); for further information and accurate numerical values please refer to Table S5 online. It is important to note that we only used top-scoring NetworKIN (Linding et al., 2007) and NetPhorest (Miller et al., 2008) predictions filtered to ensure maximum confidence, and that we reached the same conclusions using both algorithms. Because of this and the fact we have based our global observations on thousands of mutations, our conclusions drawn from Figure 5 and (B) are highly unlikely to have been affected by our choice of methods.

(B) By analyzing PSSMs characterizing the peptide specificity of a large number of human protein kinases (from the NetPhorest repository), we could quantify how much each substrate position contributes to the kinase-substrate recognition process (from seven residues before the phosphorylation position, P-7, up to seven residues after, P+7). Similar to what we observed in Figure 5 and (A), in the case of mutations hitting different positions and their likelihood to lead to upstream rewiring, position P-1 contributes relatively little to the kinase-substrate recognition process.

Cell

Supplemental Information

Kinome-wide Decoding of Network-Attacking Mutations Rewiring Cancer Signaling

Pau Creixell, Erwin M. Schoof, Craig D. Simpson, James Longden, Chad J. Miller, Hua Jane Lou, Lara Perryman, Thomas R. Cox, Nevena Zivanovic, Antonio Palmeri, Agata Wesolowska-Andersen, Manuela Helmer-Citterich, Jesper Ferkinghoff-Borg, Hiroaki Itamochi, Bernd Bodenmiller, Janine T. Erler, Benjamin E. Turk, and Rune Linding

SUPPLEMENTAL EXPERIMENTAL PROCEDURES

Maintenance of Ovarian Cancer Cell Lines and Preparation of Sequencing Samples

ES2, OVAS, OVISE, TOV-21 and KOC-7C cells were grown in RPMI supplemented with 10% FBS and 1% PenStrep. The provenance of our cell lines was confirmed by STR analysis and is further detailed in the Figure S1. DNA was extracted from 2 x 10^6 cells at 80% confluency using a Qiagen QIAamp DNA Mini kit, according to the manufacturers instructions. The purified DNA was then sent to Roche Nimblegen for full exome (SeqCap EZ Human Exome Library v3.0 capture kit) sequencing. The sequencing results (FastQ files available at the Sequence Read Archive, SRA, under the study number PRJNA248664) were aligned using the Burrows–Wheeler Alignment Tool to the human reference genome (NCBI version GRCh37). After quality score recalibration and Indel realignment using the GATK package, SNP calling was performed with SAMtools. Next, we further filtered with VCFtools' default settings as well as a minimum coverage threshold of 10X in order to obtain high-quality variants. Finally, similarly as in the case of the global repository of somatic cancer mutations, the fasta files required by ReKINect containing both wild type and mutant protein sequences for all variants were generated using the Variant Effect Predictor (VEP) (Flicek et al., 2014) and purpose-made Python scripts.

Preparation of (Phospho-)Proteomics Samples

ES2, OVAS, OVISE, TOV-21 and KOC-7C cells were labeled with medium Stable Isotope Labeling by Amino acids in cell Culture (SILAC) and grown to ~80% confluency in sufficient 15cm dishes to provide 12mg of protein as starting material in triplicate. Each medium-labeled sample would be subsequently mixed 1:1 with a Spike-in SILAC (Geiger et al., 2011; Monetti et al., 2011) sample labeled with heavy SILAC containing a mix of peptides from the different ovarian cell lines, so that such internal standard would allow inter-sample comparisons. After cell cycle synchronization, by serum starvation for 24 hours, cells were lysed using ice-cold modified RIPA buffer supplemented with Roche complete protease inhibitor cocktail tablets and ß-glycerophosphate (5mM), NaF (5mM), Na-orthovanadate (1mM, activated). After cell lysis, samples were sonicated on ice, spun down (4,400g for 20mins at 4ºC) and proteins were precipitated over-night in ice cold acetone at -20ºC. Next, samples were dissolved in 6M Urea, 2M Thiourea, 10mM HEPES pH 8.0 and further reduced with 0.1mM DTT for one hour, alkylated with 5.5mM Chloroacetamide for another hour, after which they were pre-digested with Lysyl Endopeptidase at a 1:200 enzyme-to-protein ratio for four hours at room temperature. A dilution 1:4 with 50mM Ammonium Bicarbonate followed, after which trypsin was added at a 1:200 enzyme-to-protein ratio and left rotating over-night at room temperature. The following morning, the addition of TFA to a final concentration of 2% was used to suppress the enzymatic activity of trypsin, after which the samples were clarified and desalted using 360mg SepPak columns. Peptides were eluted twice with 2ml of 40% Acetonitrile, 0.1% TFA, and one final time with 2ml of 60% Acetonitrile, 0.1% TFA. For the phospho-peptide enrichment with Titanium Dioxide (TiO_2), the eluent was directly subjected to SCX fractionation and thus separated over a 30% SCX Buffer B (5mM potassium dihydrogen phosphate, 30% Acetonitrile, 350mM potassium chloride, pH2.7) gradient in 60 minutes at a 1ml/min flowrate. The resulting fractions were pooled according to their chromatographic properties and enriched for phosphorylated peptides, while specific aliquots were taken at this point for the global proteome analysis. The TiO_2 enrichment was conducted similarly to ref (Olsen et al., 2006), with several adjustments. For the TiO_2 loading solution, the TiO_2 beads were incubated in 0.02g/ml dihydrobenzoic acid dissolved in 30% Acetonitrile and 4% TFA for 15 minutes prior to peptide enrichment. 1.5mg of TiO_2 beads suspended in 6µl of TiO_2 loading solution was subsequently used to enrich each pooled SCX fraction and left to rotate end-over-end for 30 minutes at room temperature. The flow-through (i.e. early eluting fractions) was enriched three consecutive times. Samples were spun down at 2000g for 5 minutes and pelleted beads were washed with 100µl SCX Buffer B. After spinning the samples down again as before, beads were again pelleted and washed this time with 100µl 40% Acetonitrile, 0.25% acetic acid, 0.5% TFA. Finally, pelleted beads were re-suspended in 50µl 80% Acetonitrile, 0.5% acetic acid and transferred to separate C8 StageTips. Liquid was spun through at 2000g for 60s, after which the phosphorylated peptides were eluted with 1x 20µl 5% Ammonia and 1x 20µl 10% Ammonia, 25% Acetonitrile into a 96-well PCR plate, containing 20µl of 1% TFA, 5% Acetonitrile solution. Peptides were lyophilized to a total volume of 10µl, and acidified with 40µl of 1% TFA, 5% Acetonitrile, after which they were desalted on in-house packed C18 StageTips prior to LC-MS analysis.

For LC-MS analysis, peptides were eluted from the StageTip with 2x 20µl 80% Acetonitrile, 0.1% Formic acid, and lyophilized to 5µl final volume. The eluent was acidified with 1% TFA, 2% Acetonitrile and loaded onto a 50cm C18 EasySpray column, using the EasyLC 1000 UHPLC system (Thermo Fisher Scientific, USA). Peptides were eluted over a 250 minute gradient, ranging from 6-60% of 80% Acetonitrile, 0.1% Formic acid, and the Orbitrap Q Exactive (Thermo Fisher Scientific, USA) was run in a DD-MS2 top10 method. Full MS spectra were collected at a resolution of 70,000, with an AGC target of 3 x 10^6 or maximum injection time of 20ms and a scan range of 300-1750 m/z. The MS2 spectra were obtained at a resolution of 17,500, with an AGC target value of 1 x 10^6 or maximum injection time of 80ms. Dynamic exclusion was set to 20s, and ions with a charge state < 2 or unknown were excluded. For the proteome samples, the settings were the same, except for a gradient time of 230 mins, maximum MS2 injection time of 60ms and dynamic exclusion of 45s.

As shown in Figure S2, the Spike-In approach was done using a heavy-labelled SILAC reference sample coming from a proteomic mix of all the ovarian samples. All measurements where taken in biological triplicates and comparisons between same-sample and inter-sample confirmed the robustness of our method.

The samples were analyzed by liquid chromatography-mass spectrometry on an Orbitrap Q-Exactive (Thermo Fisher Scientific, USA). The mass spectrometry proteomics data have been deposited to the ProteomeXchange Consortium (http://www.proteomexchange.org) via the PRIDE partner repository (Vizcaíno et al., 2013) with the dataset identifier PXD000901.

Computational analysis of MS spectra

In order to investigate the effect of using mutant sample-specific fasta files as the MaxQuant search engine database, we performed the raw data searches using the wild-type ENSEMBL human FASTA (GRCh37/hg19) including all missense mutant proteins reported by sequencing. Further analysis and mapping of known phosphorylation sites to detect extinction of phosphorylation sites was done using in-house Python, R and MySQL scripts. MS search results were filtered on phosphorylation localization probability higher than 0.75 and MaxQuant peptide ID score higher than 50 in order to only use high confidence identifications.

Computing Minimum Distance to Substrate from PDB Files

As discussed in the main text, we included a measure of distance to the peptide substrate in our lists of mutations perturbing determinants of specificity. This distance was extracted by computing the distance between every residue in the kinase domain to every position in the peptide substrate for ten structures deposited in PDB, where kinase and substrate were co-crystallized (AKT2 [PDB ID: 1O6K]; (Yang et al., 2002), PIM1 [PDB ID: 2BZK]; (Debreczeni Bullock, A., Knapp, S., Von Delft, F., Sundstrom, M., Arrowsmith, C., Weigelt, J., and Edwards, A.), DYRK1A [PDB ID: 2WO6]; (Soundararajan et al., 2013), CDK2 [PDB ID: 2CCI]; (Cheng et al., 2006), PAK4 [PDB ID: 2Q0N]; (Filippakopoulos Eswaran, J., Turnbull, A., Papagrigoriou, E., Pike, A.W., Von Delft, F., Sundstrom, M., Edwards, A., Arrowsmith, C.H., Weigelt, J., and Knapp, S), EPHA3 [PDB ID: 3FXX]; (Davis et al., 2009), FES [PDB ID: 3CD3]; (Filippakopoulos et al., 2008), EGFR [PDB ID: 2GS6]; (Zhang et al., 2006), IGF1R [PDB ID: 1K3A]; (Favelyukis et al., 2001), INSR [PDB ID: 3BU3]; (Wu et al., 2008)). More specifically, with the use of in-house python scripts and a biopython package (Bio.PDB), we extracted all these distance features between all residues of these kinase-substrate pairs and collapsed the information about minimum distance observed with the kinase domain alignment. Any alignment position that could not be mapped to a structure was given the arbitrary maximum distance of 50Å.

Prioritization of Cancer Somatic Mutation Most Likely Causing Downstream Rewiring.

In order to prioritize the mutations identified by ReKINect as downstream rewiring, we integrated two extra sources of information. First, to prioritize based on the distance to the substrate peptide, we collected a comprehensive set of structures of protein kinases with a bound peptide substrate deposited in Protein Data Bank (PDB) (Berman et al., 2000) and computed the minimum distance between each alignment position and the peptide substrate (see Experimental Procedures). Additionally, using the dataset collected in our accompanying article (ref. Creixell et al. co-submitted article), we utilized information about whether previous methods had already identified the positions hit by these mutations as potential DoS, essentially providing independent validation of these results.

Table S1 provides the complete prioritized list of putative downstream rewiring NAMs, ordered by the likely contribution of the site to kinase specificity as measured by KINspect (specificity score, ref. Creixell et al. co-submitted article) and, where available, other sources in the literature as well as information about the average distance of residues in this kinase position to the substrate peptide (distance to substrate in Ångstrøms, Å).

In order to identify potential candidates for experimental validation, we prioritized our mutations on DoS based on their distance to substrate, their wild-type specificity having being previously experimentally determined and independent evidence of the determinants of specificity.

Protein Kinase Specificity Assays

Briefly, we used a 182-component peptide library with the general sequence Y-A-X-X-X-X-X-S/T-X-X-X-X-A-G-K-K(biotin), in which X postions were an equimolar mix of the 17 amino acid residues (excluding Cys, Ser and Thr), and S/T was an equal mix of Ser and Thr. Each component of the library had a single X position fixed as one of the 20 amino acids. Peptide mixtures (50 μM) were arrayed in 1536 well plates and incubated with kinase and ATP (50 μM including 0.03 μCi/μl γ−[^{33}P]ATP) for 2 hr at 30 ºC. Aliquots (200 nl) were transferred to a biotin capture membrane (Promega), which was washed, dried and exposed to a phospho screen as described (Mok et al., 2010). Spot intensities were quantified (QuantityOne software, BioRad) and normalized so that the average value within a position was assigned a value of 1. Data averaged from multiple runs were $\log_2$ transformed and used to generate heat maps. For PKCγ assays on individual peptide substrates, kinase (0.1 ug/ml) was incubated with 5 μM peptide in a buffer containing 50 mM Tris-HCl, pH 7.5, 10 mM $MgCl_2$, 1 mM DTT, 100 μM ATP with 0.3 μCi/μl γ−[^{33}P]ATP, and a 5-fold dilution of PKC lipid activator (provides diacylglycerol in phosphatidylserine vesicles and calcium, EMD-Millipore) for 15 min at 30°C. Aliquots were withdrawn at 5 min intervals and analyzed by P81 filter binding assay and scintillation counting to determine radiolabel incorporation (Hastie et al., 2006). Linear reaction rates were calculated from a standard curve generated from known quantities of radiolabel.

Phenotypic RNAi screening of Mutated Kinase and SH2 proteins

Cells were transfected with Silencer Select siRNAs (Life Technologies) using a ‘one-step’ method; siRNAs were diluted to 80nM in OptiMEM (Life Technologies) and mixed 1:1 with Lipofectamine RNAiMAX, also diluted in OptiMEM, such that each siRNA was mixed with 0.08μl of reagent. The siRNA/transfection reagent mix was then incubated at room temperature for 15 minutes prior to being dispensed into 384-well ViewPlates (PerkinElmer). Cell lines were plated directly into the siRNA containing wells at a density of 2000 (ES2), 3000 (KOC7C, TOV21 and OVISE) or 4000 (OVAS) cells per well. Cells were then incubated with the siRNAs for 72 hours at 37°C, 5% CO_2, 95% humidity before being fixed, stained and read on the Opera High Content Imaging reader (PerkinElmer). siRNAs were diluted 1 in 8 by the addition of cell culture medium giving a final, ‘in-assay’ concentration of 5nM. Cells were fixed by the addition of 4% paraformaldehyde (Sigma), incubated at room temperature for 15 minutes. Parafromaldehyde was then removed and cells were stained with Hoechst 33342 (Life Technologies) diluted to 2μg/ml in PBS. Cells were incubated for 1 hour, at room temperature, in the dark before being washed and imaged. Cells were imaged on the Opera using a x10 objective, 405nm laser excitation and 450/50 band pass emission filter. Nuclei were detected using the Acapella image analysis software (PerkinElmer).

Analysis of Phenotypic Changes Culminating from Genesis and Extinction of Phosphorylation Sites

Following perhaps the most parsimonious expectation, we hypothesized that the genesis of a phosphorylation site on TANC1 could lead to an observable phenotypic readout upon its knock-down and, on the contrary, the extinction of a phosphorylation site on RAB11FIP1 could decrease any pre-existing phenotypic impact upon knock-down. While knock-down effect could certainly be attributable to many other factors besides these specific mutations, surprisingly, we indeed observed a relatively small but robust ($P=1.7x10^{-4}$) loss of nuclear intensity upon knock-down of TANC1 only in the mutant cell line, OVAS (Figure S3E). Moreover, the decreased proliferation caused by the knock-down of RAB11FIP1 in cell lines wild-type for this gene is significantly ($P=3.1x10^{-3}$) reduced compared to the cell line harboring the mutant variant, KOC7C, most likely through a perturbation of cell cycle kinetics leading to an accumulation of cells in G1-phase (Figure S3D and F).

The statistical significance of changes in cell phenotypes caused by siRNA knockdown of ReKINect classified NAMs resulting in the generation or extinction of phosphorylation sites was evaluated using 1-way fixed effect ANOVA. The measured phenotypes were related to cell proliferation and included nuclei number and nuclei intensity (which changes throughout the cell cycle and during apoptosis). This analysis showed that the KOC7C cell line had a significantly different phenotype (in terms of nuclear proliferation) from those wild type for RAB11FIP1 (ES2, OVAS, OVISE and TOV21), $P=3.1x10^{-3}$. Conversely, the OVAS cell line had a significantly different phenotype (in terms of nuclear intensity) from those wild type for TANC1 (ES2, KOC7C, OVISE and TOV21), $P=1.7x10^{-4}$.

NAM based Regressor Models of Cell Proliferation

Changes in nuclei number were assayed, as described in Experimental Procedures, utilizing a library of 573 siRNAs (Ambion Silencer Select, Life Technologies) targeting kinase and SH2 domain containing genes.

Screens were performed in the ovarian cancer cell lines ES2, KOC7C, OVAS, OVISE and TOV21, and quantified data from triplicate repeats was normalized to negative i.e. non-targeting siRNA.

Of the 2865 tested conditions 314 caused a robust (greater than 2 standard deviations from the mean of the negative control) increase in nuclei number. A further 117 caused a robust decrease in nuclei number.

From these data we set out to predict changes in proliferation, Y, upon RNAi knockdown based on the number and types of NAMs in the neighborhood of target proteins as defined by the local protein-protein interaction (PPI) network, as detailed below.

The proliferation change for a knock-down target t and cell line c is denoted as Y_{tc}. The specific measurement uncertainty of Y pertaining to any knockdown and cell line was quantified using variation from the three biological repeats, denoted as σ_{tc}. In the following subsections we shall consider four different models, described in turn.

Baseline model

It was assumed that there was no correlation between proliferation and the PPI-neighborhood of the siRNA target. Consequently the global screen is simply regressed as:

$$Y_{tc}=k+e_{tc}, \quad e_{tc}\sim N(0; \sigma_{tc})$$

where k is a common constant for all knock-downs and cell lines, representing the baseline ($k\approx100$) and e_{tc} represents the uncertainty, which is assumed to be normally distributed with a standard deviation equal to the experimentally determined one, σ_{tc}.

Topology model

We also investigated whether the network topology in the vicinity of each siRNA target had an effect on proliferation. In this model the neighborhood of each target, t, and cell line, c, was characterized in terms of the number neighbors, $n^{(d)}_{tc}$ in the PPI-network at distance d=1 and d=2. The full model reads:

$$Y_{tc}=k+\Sigma_{d=1,2}\, a_N^{(d)}\cdot N^{(d)}_{tc}+e_{tc},$$

where $N^{(d)}_{tc}$ is a standardized version of $n^{(d)}_{tc}$, to ease the biological interpretation of the corresponding coefficients. Specifically, $N^{(d)}_{tc}=(n^{(d)}_{tc}-\mu_n^{(d)})/\sigma_n^{(d)}$, where $\mu_n^{(d)}$ and $\sigma_n^{(d)}$ are respectively the mean and standard deviation of the number of proteins at distance d from target, across all targets and cell lines. Consequently, a value of $a_N^{(d)}=1$ implies that if the number of proteins at distance d is one standard deviation larger than its mean, the proliferation is predicted to be one unit larger than baseline k, given that the other regressor is set to its mean. The model potentially comprises 3 independent regression coefficients (k, $a_N^{(1)}$, $a_N^{(2)}$).

Binary model

In this model, we examined whether the presence of a NAM in the vicinity of each siRNA target had an effect on proliferation. Specifically, we combined the topology model with the number of NAMs $m^{(d)}_{tc}$, in distance d=0, 1 and 2 away from the target, ignoring uninterpreted mutations. The full model reads:

$$Y_{tc}=k+\Sigma_{d=1,2}\, a_N^{(d)}\cdot N^{(d)}_{tc}+\Sigma_{d=0,1,2}\, a_M^{(d)}\cdot M^{(d)}_{tc}+e_{tc},$$

where $M^{(d)}_{tc}$ is a standardized version of $m^{(d)}_{tc}$, , c.f. the standardization procedure for $n^{(d)}_{tc}$. The model potentially comprises 6 independent regression coefficients (k, $a_M^{(0)}$, $a_M^{(1)}$, $a_M^{(2)}$, $a_N^{(1)}$, $a_N^{(2)}$).

Classified model

Finally, to assess whether the specific NAM classification provides further predictive power we derived an extension of the Binary model in which we distinguish between different uninterpreted and interpreted mutations (NAMs).

The targets addressed in the RNAi screen were classified using ReKINect alone without prior or additional knowledge and therefore entailed only four of the possible NAM classes (*downstream rewiring*, *extinction of phosphorylation site* , *node activation* and *node inactivation*) and an 'uninterpreted' class (i.e. where a mutation is not interpreted by ReKINect alone).

These classifications were, as to be expected, very unevenly distributed (downstream rewiring = 31, extinction of phosphorylation site = 377, node inactivation = 6, node activation=1, uninterpreted = 3856). Since some of the mutation counts were too low to be informative by themselves, we grouped mutations by categories (likely signaling activating or likely signaling inactivating) by combining node inactivating mutations with phosphorylation site extinctions, and similarly, combining node activations with downstream rewiring mutation. Thus, the full model reads:

$$Y_{tc}=k+\Sigma_{d=0\text{-}2}\, a_L^{(d)}\cdot L^{(d)}_{tc}+\Sigma_{d=0\text{-}2}\, a_R^{(d)}\cdot R^{(d)}_{tc}\, \Sigma_{d=0\text{-}2}\, a_U^{(d)}\cdot U^{(d)}_{tc}+\Sigma_{d=1,2}\, a_N^{(d)}\cdot N^{(d)}_{tc}+e_{tc},$$

where $L^{(d)}_{tc}$, $R^{(d)}_{tc}$ and $U^{(d)}_{tc}$ are the standardized (see above) number of mutations classified as either a loss of a phosphorylation site, downstream rewiring or uninterpreted, respectively, as function of the cell line c and distance d from the target t. Consequently, the model potentially comprises 12 independent regression coefficients (k, $a_L^{(0,1,2)}$, $a_R^{(0,1,2)}$, $a_U^{(0,1,2)}$, $a_N^{(1)}$, $a_N^{(2)}$). The conceptual idea of the classified (and NAM) model is illustrated in Figure S3A.

PPI-network

We based the network analysis on known physical PP-interactions as indexed by the combined score in the STRING resource (Szklarczyk et al., 2011). We pruned the network by removing all links with probabilities below a predefined cut-off value P_c, and assigning probabilities P=1 to the remaining links. The regression results listed below are for P_c=0.9, corresponding to what STRING terms high-confidence. The results, however, are insensitive to this choice, as verified by repeating the analysis with other cut-off values (0.5< P_c<0.9).

Variable and model selection

In order to avoid over-fitting we pruned away non-informative regressors in each of the considered models following a 2-step procedure. In the first step, a subset of regressors were selected using two different standard methods: AIC-optimization (Akaike, 1974) and shrinkage via the elastic-net (EN) regularization (Zou and Hastie, 2005). In the first approach, the subset of variables is found by minimizing the Akaike information criterion (AIC). Due to the low-dimensional nature of the parameter space, we have determined this set by an exhaustive search of all 2^D possibilities, where D is the number of coefficients, $a_X^{(d)}$ in the model. The second approach, EN-regularization, combines two traditional shrinkage techniques, LASSO- (Tibshirani, 1994) and Tikhonov-regularization (Tikhonov and Arsenin, 1978). The method is characterized by a single global parameter f (0≤f≤1), which dictates the balance between the two types of regularization (f=0, Tikhonov and f=1, LASSO). For each value of f, we determine the overall regularization scale by minimizing the 10-fold cross-validation error using the efficient R source-package implementation "glmnet" (Friedman et al., 2010). As the cross-validation error turned out to be insensitive to the choice of f, a 'mean' EN-model was constructed by including only those regressors that appeared more than 50% of times in a scan of f from 0 to 1 in steps of 0.1.

Both the resulting AIC-model and the mean EN-model was subsequently pruned by keeping only those regressors in a standard linear regression that proved significantly different from zero (two-sided t-test, α=0.05).

The regression model with the best predictive power was then chosen as the final model.The regression results for these final versions of the NAM and classified models are given in Figure S3B, yielding a reduction to only 2 and 3 regressors, respectively, excluding the baseline.

The performance of the final classified model is illustrated in Figure S3C.

Model comparison

The four models were compared in terms of their respective AIC-score, root mean square residuals R, and P-values. AIC values for the baseline, topology model, binary model and classified model were 4606, 4587, 4554 and 4538 respectively. R values were 2.246, 2.238, 2.224 and 2.217 respectively. P-values were derived by ANOVA-model comparison.

Network topology (topology model), the presence of NAMs (binary model) and the classification of those NAMs (classified model) were all found to have significant predictive power compared to the baseline model with p-values of 4.8×10^{-6}, 7.1×10^{-13} and 6.3×10^{-16} respectively.

Cell-cycle Phase Analysis

Cell-cycle analysis by quantitation of DNA content was undertaken using flow cytometry. 72 hours after knockdown (as described in phenotypic RNAi screening of mutated Kinase and SH2 proteins, but scaled for 6-well plate format), cells were trypsinised and washed once in ice-cold phosphate-buffered saline (PBS) and counted. Cells were pelleted and then fixed by drop wise addition of ice-cold 70% ethanol while vortexing and incubated on ice for 30 minutes. Cells were then pelleted and washed twice with PBS before treatment with 50µl per 1×10^6 cells, of a 100µg/ml stock of DNase-free RNase for 15 minutes at 37'C. DNA was then stained by addition of 200µl per 1×10^6 cells, of 1µg/mL DAPI in PBS. Flow cytometry was carried out on an LSRII equipped with UV laser (BD Biosciences). Initial gating of cells was based on FSC-A and SSC-A. Doublets were excluded based on gating of FSC-A vs. SSC-W and DAPI-A vs. DAPI-W. Gated single cells were then plotted as a histogram using the DAPI-A parameter with standard gates defined for G1, S phase and G2M. Gates were defined on negative control populations and subsequently applied to corresponding knockdowns. Voltages were only adjusted between cell lines to facilitate placing of G1 populations on the same scale. All cell cycle analysis was carried out in biological triplicate for each of the cell lines and conditions.

Statistical significance of the shift in cell cycle kinetics and an accumulation of cells in G1 phase from G2/M and S phase was evaluated using a t-test. P-values for the wild type cell lines were $8.6x10^{-3}$, $1.3x10^{-2}$, $2.1x10^{-2}$ and $2.0x10^{-3}$ for ES2, OVAS, OVISE and TOV21 respectively. Conversely, the P-value for the mutant cell line, KOC7C, was $7.8x10^{-2}$ i.e. all wild type cell lines, and not the mutant cell line, showed a significant shift in cell cycle kinetics.

SUPPLEMENTAL REFERENCES

Akaike, H. (1974). A new look at the statistical model identification. IEEE Trans. Automat. Contr. *19*.

Berman, H.M., Westbrook, J., Feng, Z., Gilliland, G., Bhat, T.N., Weissig, H., Shindyalov, I.N., and Bourne, P.E. (2000). The Protein Data Bank. Nucleic Acids Res *28*, 235–242.

Cheng, K.Y., Noble, M.E., Skamnaki, V., Brown, N.R., Lowe, E.D., Kontogiannis, L., Shen, K., Cole, P.A., Siligardi, G., and Johnson, L.N. (2006). The role of the phospho-CDK2/cyclin A recruitment site in substrate recognition. J Biol Chem *281*, 23167–23179.

Davis, T.L., Walker, J.R., Allali-Hassani, A., Parker, S.A., Turk, B.E., and Dhe-Paganon, S. (2009). Structural recognition of an optimized substrate for the ephrin family of receptor tyrosine kinases. FEBS J *276*, 4395–4404.

Debreczeni Bullock, A., Knapp, S., Von Delft, F., Sundstrom, M., Arrowsmith, C., Weigelt, J., and Edwards, A., J.E. Crystal structure of the human Pim1 in complex with AMP-PNP and Pimtide.

Favelyukis, S., Till, J.H., Hubbard, S.R., and Miller, W.T. (2001). Structure and autoregulation of the insulin-like growth factor 1 receptor kinase. Nat Struct Biol *8*, 1058–1063.

Filippakopoulos, P., Kofler, M., Hantschel, O., Gish, G.D., Grebien, F., Salah, E., Neudecker, P., Kay, L.E., Turk, B.E., Superti-Furga, G., et al. (2008). Structural coupling of SH2-kinase domains links Fes and Abl substrate recognition and kinase activation. Cell *134*, 793–803.

Filippakopoulos Eswaran, J., Turnbull, A., Papagrigoriou, E., Pike, A.W., Von Delft, F., Sundstrom, M., Edwards, A., Arrowsmith, C.H., Weigelt, J., and Knapp, S, P. Structure of human p21 activating kinase 4 (PAK4) in complex with a consensus peptide.

Flicek, P., Amode, M.R., Barrell, D., Beal, K., Billis, K., Brent, S., Carvalho-Silva, D., Clapham, P., Coates, G., Fitzgerald, S., et al. (2014). Ensembl 2014. Nucleic Acids Res *42*, D749–D755.

Friedman, J., Hastie, T., and Tibshirani, R. (2010). Regularization Paths for Generalized Linear Models via Coordinate Descent. J. Stat. Softw. *33*, 1–22.

Geiger, T., Wisniewski, J.R., Cox, J., Zanivan, S., Kruger, M., Ishihama, Y., and Mann, M. (2011). Use of stable isotope labeling by amino acids in cell culture as a spike-in standard in quantitative proteomics. Nat Protoc *6*, 147–157.

Hastie, C.J., McLauchlan, H.J., and Cohen, P. (2006). Assay of protein kinases using radiolabeled ATP: a protocol. Nat. Protoc. *1*, 968–971.

Mok, J., Kim, P.M., Lam, H.Y.K., Piccirillo, S., Zhou, X., Jeschke, G.R., Sheridan, D.L., Parker, S.A., Desai, V., Jwa, M., et al. (2010). Deciphering protein kinase specificity through large-scale analysis of yeast phosphorylation site motifs. Sci Signal *3*, ra12.

Monetti, M., Nagaraj, N., Sharma, K., and Mann, M. (2011). Large-scale phosphosite quantification in tissues by a spike-in SILAC method. Nat Meth *8*, 655–658.

Olsen, J. V, Blagoev, B., Gnad, F., Macek, B., Kumar, C., Mortensen, P., and Mann, M. (2006). Global, in vivo, and site-specific phosphorylation dynamics in signaling networks. Cell *127*, 635–648.

Soundararajan, M., Roos, A.K., Savitsky, P., Filippakopoulos, P., Kettenbach, A.N., Olsen, J. V, Gerber, S.A., Eswaran, J., Knapp, S., and Elkins, J.M. (2013). Structures of Down syndrome kinases, DYRKs, reveal mechanisms of kinase activation and substrate recognition. Structure *21*, 986–996.

Szklarczyk, D., Franceschini, A., Kuhn, M., Simonovic, M., Roth, A., Minguez, P., Doerks, T., Stark, M., Muller, J., Bork, P., et al. (2011). The STRING database in 2011: Functional interaction networks of proteins, globally integrated and scored. Nucleic Acids Res. *39*.

Tibshirani, R. (1994). Regression Selection and Shrinkage via the Lasso. J. R. Stat. Soc. B *58*, 267–288.

Tikhonov, A.N., and Arsenin, V.Y. (1978). Solutions of Ill-Posed Problems. Math. Comput. *32*, 1320–1322.

Vizcaíno, J.A., Côté, R.G., Csordas, A., Dianes, J.A., Fabregat, A., Foster, J.M., Griss, J., Alpi, E., Birim, M., Contell, J., et al. (2013). The Proteomics Identifications (PRIDE) database and associated tools: status in 2013. Nucleic Acids Res. *41*, D1063–D1069.

Wu, J., Tseng, Y.D., Xu, C.F., Neubert, T.A., White, M.F., and Hubbard, S.R. (2008). Structural and biochemical characterization of the KRLB region in insulin receptor substrate-2. Nat Struct Mol Biol *15*, 251–258.

Yang, J., Cron, P., Good, V.M., Thompson, V., Hemmings, B.A., and Barford, D. (2002). Crystal structure of an activated Akt/protein kinase B ternary complex with GSK3-peptide and AMP-PNP. Nat Struct Biol *9*, 940–944.

Zhang, X., Gureasko, J., Shen, K., Cole, P.A., and Kuriyan, J. (2006). An allosteric mechanism for activation of the kinase domain of epidermal growth factor receptor. Cell *125*, 1137–1149.

Zou, H., and Hastie, T. (2005). Regularization and variable selection via the elastic net. J. Royal Stat. Soc. B *67*, 301–320.
